# Psychosis Risk Candidate ZNF804A Localizes to Synapses and Regulates Neurite Formation and Dendritic Spine Structure

**DOI:** 10.1016/j.biopsych.2016.08.038

**Published:** 2017-07-01

**Authors:** P.J. Michael Deans, Pooja Raval, Katherine J. Sellers, Nicholas J.F. Gatford, Sanjay Halai, Rodrigo R.R. Duarte, Carole Shum, Katherine Warre-Cornish, Victoria E. Kaplun, Graham Cocks, Matthew Hill, Nicholas J. Bray, Jack Price, Deepak P. Srivastava

**Affiliations:** a Department of Basic and Clinical Neuroscience, Maurice Wohl Clinical Neuroscience Institute, London; b MRC Centre for Neurodevelopmental Disorders, Institute of Psychiatry, Psychology and Neuroscience, King’s College London, London; United Kingdom; c MRC Centre for Neuropsychiatric Genetics & Genomics, Cardiff, United Kingdom; d Neuroscience and Mental Health Research Institute, College of Biomedical and Life Sciences, Cardiff University School of Medicine, Cardiff University, Cardiff, United Kingdom

**Keywords:** Autism spectrum disorder, Bipolar disorder, Dendritic spine, GluN1, Human neurons, Induced pluripotent stem cells (iPSCs), Neural progenitor cells, PSD-95, Psychosis, Schizophrenia, Super-resolution microscopy, Synapse

## Abstract

**Background:**

Variation in the gene encoding zinc finger binding protein 804A (*ZNF804A*) is associated with schizophrenia and bipolar disorder. Evidence suggests that ZNF804A is a regulator of gene transcription and is present in nuclear and extranuclear compartments. However, a detailed examination of ZNF804A distribution and its neuronal functions has yet to be performed.

**Methods:**

The localization of ZNF804A protein was examined in neurons derived from human neural progenitor cells, human induced pluripotent stem cells, or in primary rat cortical neurons. In addition, small interfering RNA-mediated knockdown of ZNF804A was conducted to determine its role in neurite formation, maintenance of dendritic spine morphology, and responses to activity-dependent stimulations.

**Results:**

Endogenous ZNF804A protein localized to somatodendritic compartments and colocalized with the putative synaptic markers in young neurons derived from human neural progenitor cells and human induced pluripotent stem cells. In mature rat neurons, Zfp804A, the homolog of ZNF804A, was present in a subset of dendritic spines and colocalized with synaptic proteins in specific nanodomains, as determined by super-resolution microscopy. Interestingly, knockdown of ZNF804A attenuated neurite outgrowth in young neurons, an effect potentially mediated by reduced neuroligin-4 expression. Furthermore, knockdown of ZNF804A in mature neurons resulted in the loss of dendritic spine density and impaired responses to activity-dependent stimulation.

**Conclusions:**

These data reveal a novel subcellular distribution for ZNF804A within somatodendritic compartments and a nanoscopic organization at excitatory synapses. Moreover, our results suggest that ZNF804A plays an active role in neurite formation, maintenance of dendritic spines, and activity-dependent structural plasticity.

The single nucleotide polymorphism rs1344706 within the second intron of *ZNF804A* was the first to show genome-wide significant association for the broad psychosis phenotype (1). Subsequent studies have confirmed association between common variants in *ZNF804A* and both schizophrenia (SCZ) and bipolar disorder (BP) (2, 3). In addition, copy number variations and chromosomal abnormalities affecting the *ZNF804A* gene have also been identified in cases of SCZ, BP, anxiety disorders, and autism spectrum disorders (ASDs) (4, 5, 6, 7). Whereas the rs1344706 single nucleotide polymorphism has been associated with brain function and structure (8, 9), relatively little is known about the cellular functions of zinc finger binding protein 804A (ZNF804A).

In silico modeling has revealed that *ZNF804A* encodes a protein containing a C2H2-type zinc-finger domain (10), a motif best known for binding DNA and regulating transcription (11). Several splice variants of *ZNF804A* have also been identified (10), including *ZNF804A*^*E3/E4*^, which has been reported to show reduced expression in patients with SCZ (12), and a transcript containing an alternative exon, termed exon 2.2 (13) (Supplemental Figure S1A).

Two independent studies have shown that the risk allele of rs1344706 is associated with reduced expression of *ZNF804A* RNA in the human fetal brain (12, 14). Knockdown of *ZNF804A* in human neural progenitor cells (hNPCs) or in developing neurons derived from human induced pluripotent stem cells (hiPSC-neurons) results in altered expression of genes involved in cell adhesion, neurite outgrowth, and synapse formation, as well as cytokine signaling (15, 16). Similarly, overexpression of ZNF804A in rat neural progenitor cells (NPCs) has been reported to alter the expression of several genes associated with SCZ (17). This latter study further localized the rat homologue of ZNF804A, Zfp804A, to the nucleus of rat NPCs (17). More recently, ZNF804A/Zfp804A has also been found along neurites of developing rat neurons (18) and in extranuclear compartments in adult human neurons (12). However, a detailed characterization of ZNF804A/Zfp804A subcellular distribution has not been performed to date. Critically, the role of this protein in regulating neuronal morphology and function has yet to be determined.

In this study, we demonstrate that ZNF804A localizes to nuclear and extranuclear compartments in hNPCs and is found at putative synapses in neurons differentiated from hNPCs or hiPSCs. With the use of a small interfering RNA (siRNA)-based approach, we find that loss of ZNF804A attenuates the development of early neuronal morphology. Moreover, we show that ZNF804A knockdown results in reduced expression of the adhesion protein neuroligin-4 (NLGN4) and that overexpression of NLGN4 rescues aberrant neurite outgrowth in ZNF804A knockdown neurons. In mature neurons, super-resolution microscopy reveals that Zfp804A is located at the base of spines and at perisynaptic domains within the spine head. Here, Zfp804A is present in distinct nanodomains that contain the synaptic protein postsynaptic density (PSD)-95 and *N*-methyl-D-aspartate (NMDA) receptor subunits. Finally, knockdown of Zfp804A in mature cortical neurons causes a loss of dendritic spine density and impairs activity-dependent structural plasticity. These data reveal a novel subcellular distribution for ZNF804A/Zfp8084A as well as determining a functional role in the formation of early neuronal morphology and the regulation of excitatory synapses.

## Methods and Materials

See the Supplement for further detailed Methods and Materials.

### CTX0E16 hNPCs and Neurons

Experiments using hNPCs were carried out using the CTX0E16 neural cell line obtained from ReNeuron Ltd. (www.reneuron.com) under a Material Transfer Agreement. This karyotypically normal hNPC line was derived from 12-week fetal cortical neuroepithelium and conditionally immortalized using a c-mycER^TAM^ transgene (19, 20). Proliferation and neuralization of CTX0E16 cells were carried out as previously described (19). Briefly, neuralization was achieved by replacing Dulbecco’s Modified Eagle Medium with Ham’s F-12 with Neurobasal Medium supplemented with B27 supplement (Life Technologies, Paisley, United Kingdom).

### Structured Illumination Microscopy

Structured illumination microscopy (SIM) imaging was carried out on primary rat cortical neurons transfected with green fluorescent protein (GFP) and coimmunostained for ZNF804A (GeneTex, Irvine, CA) and either PSD-95 or NMDA receptor subunit 1 (GluN1). Images were acquired with a Nikon (Kingston, United Kingdom) N-SIM super-resolution microscope. Acquisition was performed in a 3D SIM mode using the 100× 1.49 NA total internal reflection fluorescence objective lens with 13 to 25 *z* steps per stack at a step interval of 0.12 µm. Image reconstructions were performed with Nikon N-SIM software. Final images, including *z* projections and brightness and contrast adjustments, were carried out in ImageJ (National Institutes of Health, Bethesda) and MetaMorph (Molecular Devices, Sunnyvale, CA).

## Results

### Subcellular Distribution of ZNF804A in hNPCs and Neurons

**Figure 1:**
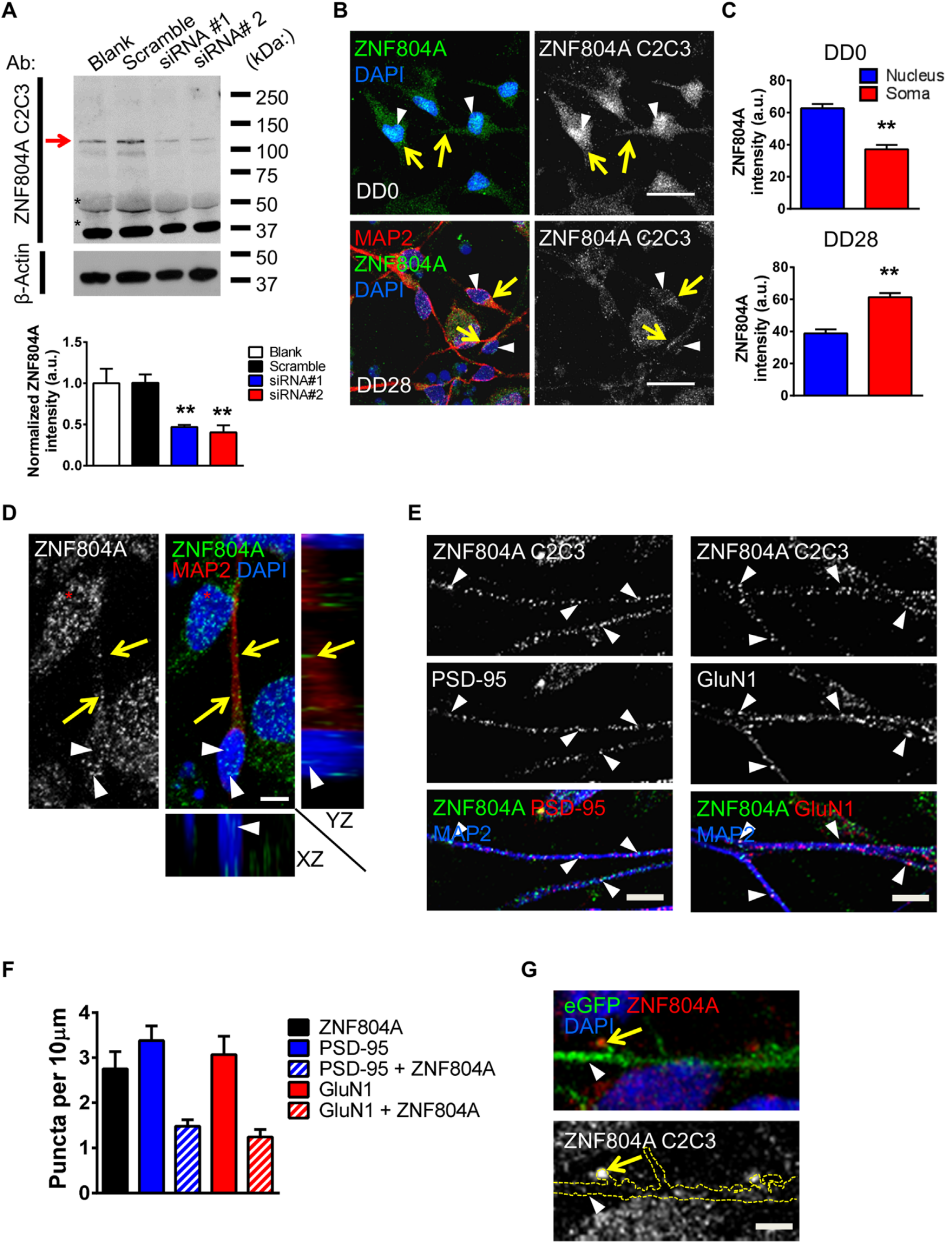
Extranuclear distribution of zinc finger binding protein 804A (ZNF804A) in CTX0E16 cells. **(A)** Western blot of ZNF804A and quantification of knockdown after 7-day small interfering RNA (siRNA) treatment in early stage CTX0E16 cells using C2C3 anti-ZNF804A antibody. Expression of the major protein band at ~135 kDa (indicated by red arrow) is significantly reduced in the presence of siRNA 1 and 2 (*n* = 3 independent experiments); asterisks indicate nonspecific bands. **(B)** Distribution of endogenous ZNF804A in proliferative (DD0) and differentiated (DD28) CTX0E16 cells as determined by immunocytochemical analysis using C2C3 antibody. ZNF804A immunoreactive puncta were observed within the nucleus (white arrowheads), soma, and in neurite-like protrusions (yellow arrows). **(C)** Quantification of relative staining intensity of ZNF804A in nuclei and cytoplasm of DD0 and DD28 CTX0E16 cells (*n* = 3 independent experiments). **(D)** In DD28 CTX0E16 neurons ZNF804A was localized to the nucleus (white arrowheads) and along microtubule-associated protein 2 (MAP2)-positive dendrites (yellow arrows) using the GeneTex anti-ZNF804A antibody. Orthogonal projections confirm ZNF804A presence at nuclear and extranuclear sites. **(E)** ZNF804A is present along distal dendrites of DD28 CTX0E16 neurons, where a subset of puncta colocalize with putative synaptic markers. White arrowheads indicate colocalization of ZNF804A with postsynaptic density (PSD)-95 or the *N*-methyl-D-aspartate (NMDA) receptor subunit 1 (GluN1). **(F)** Quantification of ZNF804A, PSD-95, GluN1, and colocalized puncta density in DD28 CTX0E16 neurons (puncta per 10 µm: ZNF804A, 2.8 ± 0.38; PSD-95, 3.4 ± 0.32; GluN1, 3.1 ± 0.4; ZNF804A + PSD-95, 1.5 ± 0.14; ZNF804A + GluN1, 1.2 ± 0.17; *n* = 17 neurons from three independent experiments). **(G)** ZNF804A puncta were present within the heads of dendritic spines of DD35 CTX0E16 neurons transfected with enhanced green fluorescent protein (eGFP). Yellow arrows indicate ZNF804A puncta within the heads of dendritic spines, white arrowheads indicate puncta within the dendritic shaft. Scale bars = 20 µm **(B)**, 5 µm **(D, E, G)**. Ab, antibody; a.u., arbitrary unit; DAPI, 4′,6-diamidino-2-phenylindole. **p < .05 **(A)**, **p < .01 **(C)**.

We first examined the subcellular distribution of ZNF804A in a conditionally immortalized human neural cell line, CTX0E16 (19). Polymerase chain reaction analysis of oligo(dT)-primed complementary DNA showed expression of ZNF804A exon 4 [included in *ZNF804A* variant b (10) and E2E3 (12) as well as full-length ZNF804A transcripts] in proliferating CTX0E16 cells and during their early neuronal differentiation (Supplemental Figure S1B). Expression of exon 2.2 (13) and E2E3 (12) was also detected in these cells at the assayed time points using transcript-specific primers (Supplemental Figure S1B). However, quantitative polymerase chain reaction analysis revealed that transcripts probed by *ZNF804A*^*exon4*^ primers were far more abundant than *ZNF804A*^*E3/E4*^ alone in both proliferative and differentiated CTX0E16 cells (Supplemental Figure S1C). Interestingly, *ZNF804A*^*exon4*^ levels were significantly reduced as CTX0E16 NPCs differentiated into neurons, whereas *ZNF804A*^*E3/E4*^ expression was unaltered (Supplemental Figure S1C). To examine the distribution of ZNF804A we used an antibody raised against the C-terminal region (C2C3; GeneTex). This antibody detected a major protein band at ~135 kDa corresponding to full-length ZNF804A in days differentiated (DD) 35 CTX0E16-neuronal lysates (see Supplemental Figures S1–S5 and Supplemental Results for additional information). Importantly, two previously characterized siRNAs targeting ZNF804A (15) specifically reduced this major protein band (Figure 1A), indicating that this antibody is specific for full-length ZNF804A; both siRNAs also reduced *ZNF804A*^*exon4*^ messenger RNA expression (Supplemental Figure S1H).

With the use of the C2C3 antibody, we examined the cellular distribution of ZNF804A in CTX0E16 hNPC proliferative and differentiated (DD28) neurons. ZNF804A was present in the nuclei of hNPCs and DD28 neurons (Figure 1B) but was also observed in the soma and processes of hNPCs and along microtubule-associated protein 2 (MAP2)-positive dendrites in DD28 neurons (Figure 1B). A similar distribution was also seen in DD10 and DD15 CTX0E16 neurons (Supplemental Figure S4A). In CTX0E16 NPCs and hEK293 cells, exogenously expressed GFP-ZNF804A also localized to the nucleus, soma, and at the plasma membrane (Supplemental Figure S4B). Exogenously expressed myc-ZNF804A in early stage CTX0E16 neurons also localized along double cortin-positive neurites further supporting an extranuclear distribution in neurons (Supplemental Figure S4C). Furthermore, expression of a Halo-tagged ZNF804A construct in SH-SY5Y cells also displayed an extranuclear distribution in both live and fixed cells (Appendix A, Appendix A). Interestingly, ZNF804A staining was more abundant in the nucleus compared with the soma in CTX0E16 NPCs. Conversely, in CTX0E16 neurons ZNF804A immunoreactivity was greater in the soma than in the nucleus (Figure 1C), indicating that the distribution of the protein changed over time with the development of a neuronal morphology. Collectively, these data indicate that ZNF804A is predominately present in the nucleus of hNPCs but is more abundant in extranuclear locations in neurons.

### ZNF804A Is Present Along Dendrites and Colocalizes With Synaptic Proteins

Examination of DD28 CTX0E16 neurons revealed ZNF804A immunoreactive staining as punctate structures in the nucleus, in the cell soma, and along MAP2-positive dendrites (Figure 1, Figure 1). In distal MAP2-postive dendrites, a subpopulation of ZNF804A puncta (~44%) colocalized with the prototypical synaptic marker, PSD-95 (Figure 1, Figure 1). Moreover, ~40% of ZNF804A puncta colocalized with the NMDA receptor subunit, GluN1 (Figure 1, Figure 1). In DD35 CTX0E16 neurons expressing enhanced GFP, ZNF804A puncta were observed along dendrites as well as within filopodia-like dendritic protrusions, further suggesting that the protein is targeted to postsynaptic compartments (Figure 1G). These data suggest that ZNF804A is targeted to dendrites and, furthermore, partially localizes to putative synapses in developing neurons.

### ZNF804A Regulates Early Neurite Formation

**Figure 2:**
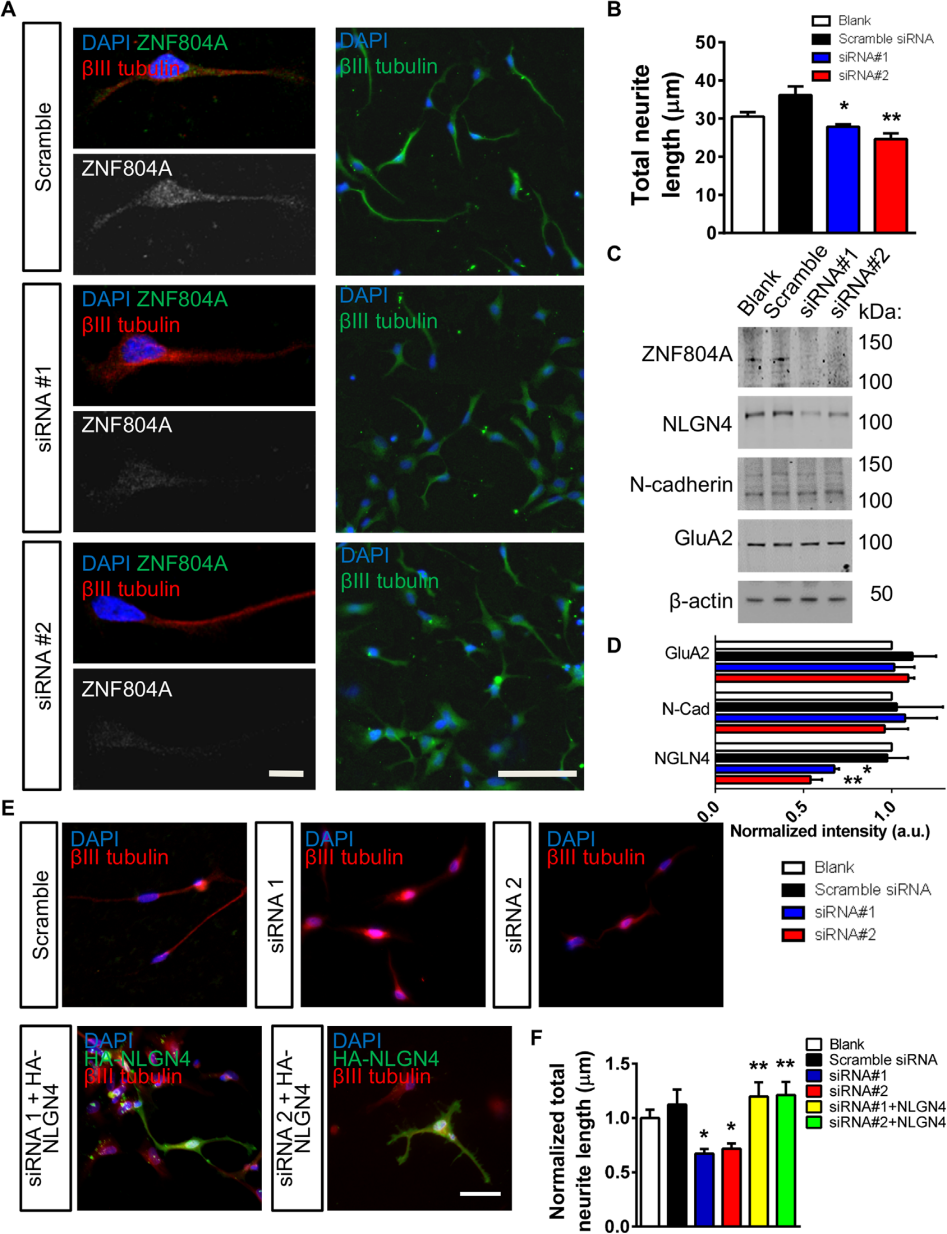
Small interfering RNA (siRNA)-mediated knockdown of zinc finger binding protein 804A (ZNF804A) in CTX0E16 neurons. **(A)** Representative images of ZNF804A staining and neurite length in early stage (DD7) CTX0E16 neurons after a 7-day treatment with no transfection (blank), control (scramble), or ZNF804A-targeting siRNAs. **(B)** Total neurite length was significantly reduced in neurons treated with both siRNAs relative to the scramble siRNA control (*n* = 3 independent experiments). **(C, D)** Western blot analysis of synaptic and adhesion protein expression in control and siRNA expressing CTX0E16 neurons. In ZNF804A knockdown cells, neuroligin-4 (NLGN4) is significantly reduced; N-cadherin and AMPA receptor subunit 2 (GluA2) are unaffected. **(E)** Representative images of early stage (DD7) CTX0E16 neurons after a 7-day treatment with no transfection (blank), control (scramble), or ZNF804A-targeting siRNAs, with or without exogenous expression of HA-NLGN4 for 2 days. **(F)** Quantification of neurite outgrowth of cells in panel E. siRNA-treated cells had significantly reduced outgrowth compared with blank or scramble conditions. Neurons treated with ZNF804A-siRNA and HA-NLGN4 had significantly longer neurites than siRNA alone conditions, but they did not differ from control conditions (*n* = 3–4 independent experiments). Scale bars = 10 µm **(A1)**, 50 µm **(A2)**, and 20 µm **(E)**. AMPA, alpha-amino-3-hydroxy-5-methyl-4-isoxazole propionic acid; a.u., arbitrary unit; DAPI, 4′,6-diamidino-2-phenylindole; N-cad, N-cadherin. *p < .05, **p < .01.

Because previous studies have suggested that ZNF804A regulates the expression of genes involved in cell adhesion, neurite outgrowth, and synaptic transmission (15, 19), we reasoned that ZNF804A might influence the development of early neuronal morphology. With the use of siRNAs, we reduced ZNF804A expression in CTX0E16 neurons and examined neurite outgrowth after 7 days of differentiation. ZNF804A protein levels were reduced by between 30% and 40% by siRNA 1 and siRNA 2, respectively (Figure 1A and Supplemental Figures S1H and 3A–C). Measurements of neuronal morphology revealed that total neurite length was significantly reduced in the presence of ZNF804A knockdown (total neurite length: scramble siRNA, 36.1 ± 1.9 µm; siRNA 1, 27.8 ± 0.5 µm; siRNA 2, 24.6 ± 1.3 µm; Figure 2, Figure 2). ZNF804A has been suggested to control the expression of *NLGN4X* (16), a member of the neuroligin-neurexin family of adhesion proteins. This protein is associated with ASDs, and it localizes to and regulates synapse density (21, 22). Intriguingly, we observed a significant reduction in NLGN4 levels in cell lysates from ZNF804A siRNA CTX0E16 neurons; the adhesion protein N-cadherin and GluA2–alpha-amino-3-hydroxy-5-methyl-4-isoxazole propionic acid (AMPA) receptor subunit were unaffected (Figure 2C). NLGN4 has been suggested to be involved in neurite outgrowth (23); however, this has not been validated. Nevertheless, we reasoned that if NLGN4 was important for neurite outgrowth, exogenous expression of this protein would rescue deficits in neurite length in siRNA-treated cells. Consistent with this, total neurite length in siRNA 1- or 2-treated cells, overexpressing HA-NLGN4, was significantly increased compared with siRNA-alone conditions but did not differ from control conditions (Figure 2, Figure 2). Collectively, these data demonstrate that ZNF804A plays a role in early neuritogenesis in human neurons, an effect that may be mediated via the control of NLGN4 expression.

### ZNF804A Localizes to Synapses and Regulates Neuritogenesis in hiPSC Neurons

**Figure 3:**
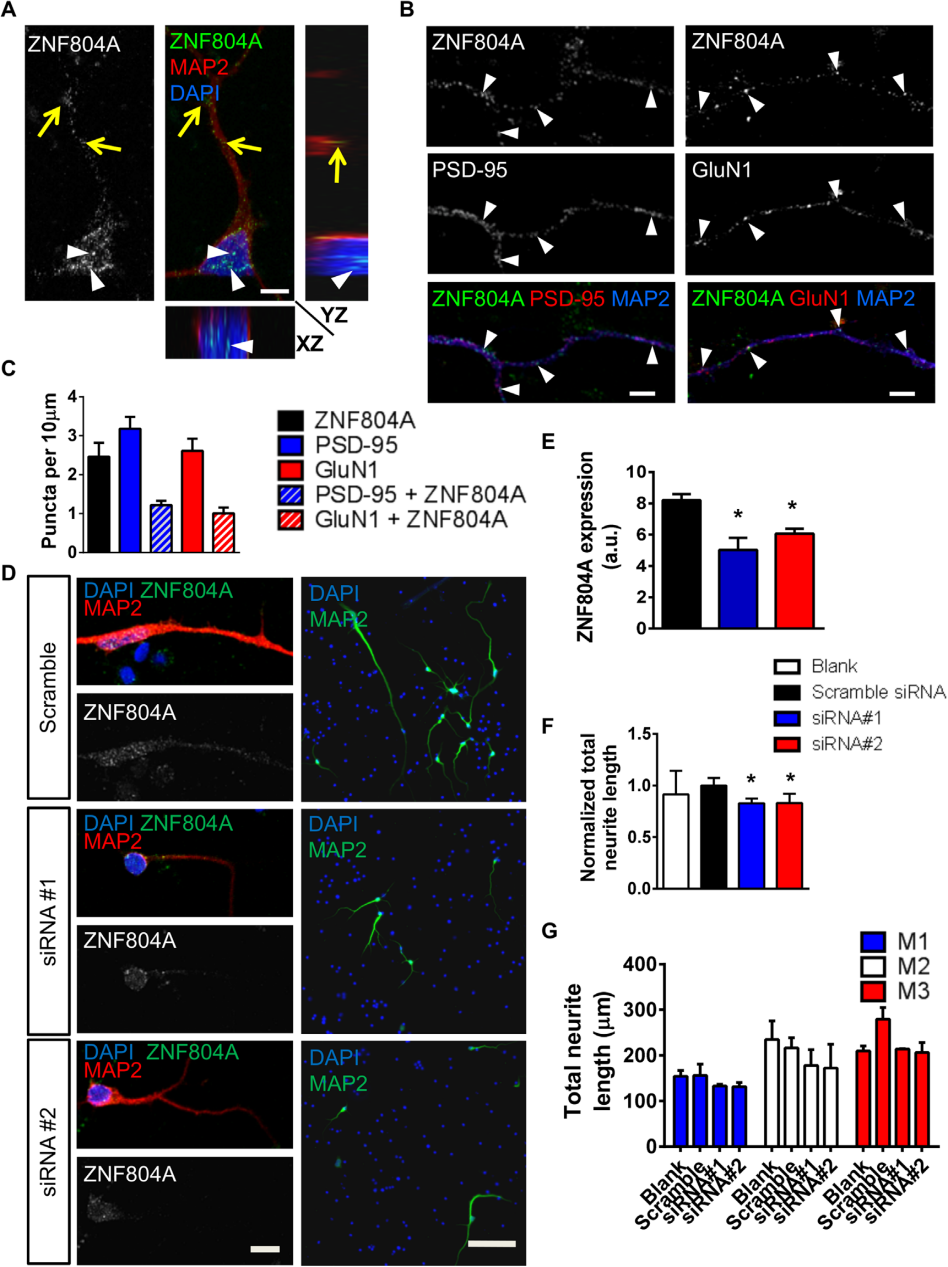
Localization and small interfering RNA (siRNA)-mediated knockdown of zinc finger binding protein 804A (ZNF804A) in young human induced pluripotent stem cell (hiPSC)-derived neurons. **(A)** In 35-day-old neurons differentiated from hiPSCs, ZNF804A puncta were observed in the nucleus (white arrowheads) and along dendrites (yellow arrows) in XY, XZ, and YZ planes. **(B)** ZNF804A puncta are also found along distal microtubule-associated protein 2 (MAP2)-positive dendrites. Costaining with postsynaptic density (PSD)-95 or NMDA receptor subunit 1 (GluN1) reveals that a fraction of ZNF804A puncta colocalize with either synaptic protein. **(C)** Quantification of ZNF804A, PSD-95, GluN1, and colocalized puncta in hiPSC neurons (puncta per 10 µm: ZNF804A, 2.5 ± 0.36; PSD-95, 3.2 ± 0.3; GluN1, 2.6 ± 0.32; ZNF804A + PSD-95, 1.2 ± 0.11; ZNF804A + GluN1, 1.0 ± 0.15; *n* = 16 neurons from three independent experiments). **(D)** Representative images of ZNF804A staining and neurite length in early stage (day [D] 30) hiPSC-derived neurons after a 7-day treatment with no transfection (blank), control (scramble), or ZNF804A-targeting siRNAs. **(E)** Quantification of staining intensity seen in panel D. ZNF804A expression was significantly reduced in both siRNA treatment conditions relative to the scramble siRNA control (*n* = 3 independent cell lines, carried out in triplicate for each line). **(F)** Normalized total neurite length was significantly reduced in neurons treated with both siRNAs relative to blank and scramble siRNA control (*n* = 3 independent cell lines, carried out in triplicate for each line). **(G)** Raw total neurite length data for siRNA-treated neurons from each hiPSC line. Scale bars = 5 µm **(A, B)**, 10 µm **(D1)**, and 50 µm **(D2)**. a.u. arbitrary unit; DAPI, 4′,6-diamidino-2-phenylindole; M1, subject 1; M2, subject 2; M3, subject 3. *p < .05.

HiPSCs provide a novel cellular system in which to confirm and elucidate the impact of genetic variations on cellular phenotypes (24, 25, 26). We were therefore interested in determining whether the distribution of ZNF804A and its purported role in neuritogenesis could be recapitulated in hiPSC neurons. HiPSCs were differentiated using a protocol that robustly generates forebrain excitatory neurons (Supplemental Figure S6A–E) (27, 28). In day (D) 35 hiPSC neurons ZNF804A was detected in the nucleus, in the soma, and along the dendrites of MAP2-positive hiPSC neurons (Figure 3A). ZNF804A was also observed as punctate structures along distal portions of MAP2-positive dendrites (Figure 3B). As with CTX0E16 neurons, a subset of ZNF804A puncta (~39%) colocalized with PSD-95 (Figure 3, Figure 3) and approximately 45% colocalized with GluN1 (Figure 3, Figure 3).

To confirm a role for ZNF804A in neuritogenesis, we knocked down ZNF804A using siRNAs in hiPSC neurons derived from three individuals (M1, M2, and M3); cells were transfected on D23 and fixed 7 days later (D30). Knockdown of ZNF804A was confirmed by immunocytochemical analysis: siRNA 1 and siRNA 2 reduced ZNF804A protein levels by between 30% and 40% (Figure 3, Figure 3). Assessment of neurite outgrowth across all three lines revealed a significant reduction in total neurite length in hiPSC neurons transfected with either siRNA 1 or siRNA 2 (normalized neurite length: blank, 0.91 ± 0.09 µm; scramble siRNA, 1 ± 0.07 µm; siRNA 1, 0.82 ± 0.04 µm; siRNA 2, 0.83.6 ± 0.08 µm; Figure 3D–G). Taken together, these data demonstrate an extranuclear distribution for ZNF804A in young human neurons and, furthermore, indicate that at early stages of neuronal development a subpopulation of the protein is present at putative synapses in human neurons. Moreover, these data further support a critical role for ZNF804A in neuritogenesis in young human neurons.

### Zfp804A Localizes to Synapses in Neurons With a Mature Morphology

**Figure 4:**
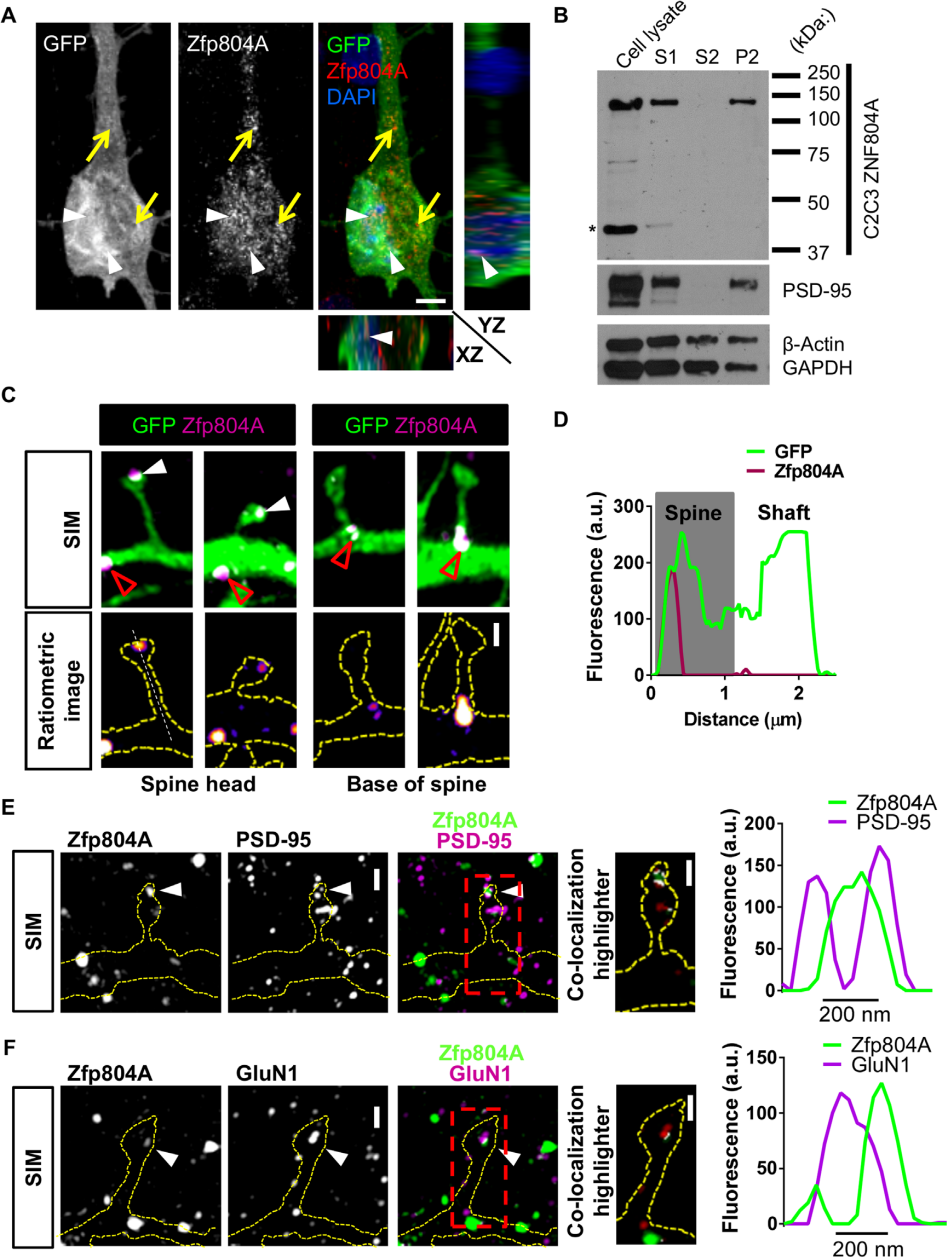
Zinc finger binding protein 804A (Zfp804A) is present at synapses in mature primary cortical neurons. **(A)** Representative confocal image, including orthogonal views in the XZ and YZ planes, of a 25-day in vitro (DIV25) rat primary cortical neuron transfected with enhanced green fluorescent protein (GFP) and immunostained for endogenous Zfp804A (rodent homologue of ZNF804A). Expression of Zfp804A within the cell nucleus is indicated with white arrowheads, and expression of Zfp804A within the cell soma and in proximal dendrites is indicated by yellow arrows. **(B)** Subcellular fractionation of DIV25 rat primary cortical neurons. Note protein band around the predicted size of 137 kDa for Zfp804A within cell lysate, post-nuclear fraction (S1), and crude synaptosomal fraction (P2), but not crude cytosolic fraction (S2), consistent with an enrichment of Zfp804A at synapses. **(C)** Super-resolution structured illumination microscopy (SIM) images of Zfp804A subsynaptic localization in primary cortical neurons. Zfp804A is present in spine heads (white arrowheads) and along the dendrite (red arrowheads). Outline of neuronal morphology is indicated by yellow dashed lines. Zfp804A signal could also be detected at the base of spines (white arrowheads) and occasionally in spine necks (red open arrowheads). **(D)** Line scan analysis through the spine and shaft shows that Zfp804A is enriched within the heads of spines. **(E, F)** High magnification SIM image of colocalized Zfp804A and **(E)** postsynaptic density (PSD)-95 or **(F)** NMDA receptor subunit 1 (GluN1) puncta (indicated with red triangles) within GFP-transfected dendritic spines (outline of spine indicated with yellow dashed lines); colocalization is indicated by white overlap. Intensity profiles of colocalized Zfp804A and either PSD-95 or GluN1 within spine heads demonstrates partial colocalization of the puncta. Scale bars = 5 µm **(A)**, 1 µm **(C)**, and 500 nm **(E, F)**. a.u., arbitrary unit; DAPI, 4′,6-diamidino-2-phenylindole; GAPDH, glyceraldehyde-3-phosphate dehydrogenase.

Because ZNF804A has been shown to be present in adult human pyramidal neurons (12, 29), and because our data suggest that ZNF804A is present at putative synapses in young neurons, we postulated that this protein may also be present at synaptic sites in mature neurons. Thus, we examined the distribution of Zfp804A in primary rat cortical neurons. Zfp804A was found to localize to nuclear and somatodendritic compartments of cortical neurons (Figure 4A and Supplemental Figure S7A). Along distal dendrites, Zfp804A localized to dendritic shafts and in dendritic spines (Appendix A, Appendix A): 44.5% ± 5.9% of spines were positive for Zfp804A. Because spine morphology is thought to be a critical indicator of synaptic function (30, 31), we classified dendritic spines that contained Zfp804A according to dendritic spine area. Of spines that contained Zfp804A, the majority (71.6%) of spines had an area less than 1.0 µm^2^ (Supplemental Figure S7D). Interestingly, Zfp804A puncta were also observed along axon-like processes, indicating a potential localization at presynaptic sites (Supplemental Figure S8A). Consistent with these immunostaining data, Western blot analysis of crude synaptosomal preparations revealed a significant enrichment of Zfp804A in synaptic lysates (Figure 4B). Taken together, these data indicate that Zfp804A is present within the dendritic shaft and in presynaptic and postsynaptic structures in mature neurons.

### Subsynaptic Localization and Organization of Zfp804A

There is now a growing appreciation that proteins within dendritic spines are organized into subsynaptic domains (32, 33); the localization of these domains plays a critical role in determining the precise functional role these proteins may play (34, 35, 36). Therefore, we were interested in the subsynaptic localization of Zfp804A within dendritic spines, because it may provide additional insights into its role at synapses. Because the resolution of traditional confocal microscopy is limited to ~200 nm, we used SIM, a form of super-resolution microscopy that exceeds this diffraction limit (37). With the use of SIM we found that Zfp804A was located in nanoscopic structures along dendrites and in spines (Supplemental Figure S8B) and axon-like processes (Supplemental Figure S8C). SIM imaging further revealed that Zfp804A was distributed into small nanoscopic domains within spine heads (Figure 4C); line scan analysis further confirmed the enrichment of Zfp804A within spine heads (Figure 4, Figure 4). Zfp804A also localized to distinct dendritic compartments and was present at the base of dendritic spines (Figure 4C). Collectively, these data indicate that Zfp804A is present at discrete nanoscopic compartments in presynaptic and postsynaptic structures, and, moreover, is distributed into small subsynaptic domains within spines.

The organization of proteins within the PSD is critical for the function of the synapse. Ultrastructural studies have shown that both PSD-95 and GluN1 are core components of the PSD (38). Given our data showing Zfp804A can be located in spine heads (Figure 4C), we hypothesized that Zfp804A also colocalize with synaptic proteins within spines. Confocal imaging revealed that ~52% of Zfp804A-positive spines contained PSD-95 and that ~43% contained GluN1 (Appendix A, Appendix A). A subset of Zfp804A puncta also colocalize with the presynaptic marker bassoon in mouse cortical sections (Supplemental Figure S9A, B). SIM imaging revealed a nonuniform distribution of Zfp804A within spine heads that was surrounded by, and only partially colocalized with, PSD-95. Intensity profiles across colocalized Zfp804A and PSD-95 in spines indicated a partial colocalization of the two proteins (Figure 4E and Supplemental Figure S9C). Interestingly, Zfp804A and GluN1 partially colocalized within spine heads; this distribution was confirmed by intensity profiles of colocalized puncta (Figure 4F and Supplemental Figure S9D). Taken together, these data suggest that within spine heads Zfp804A predominately displays a perisynaptic localization, with a small amount of protein potentially present within the PSD.

### Zfp804A Is Required for the Maintenance of Dendritic Spines and Activity-Dependent Structural Plasticity

**Figure 5:**
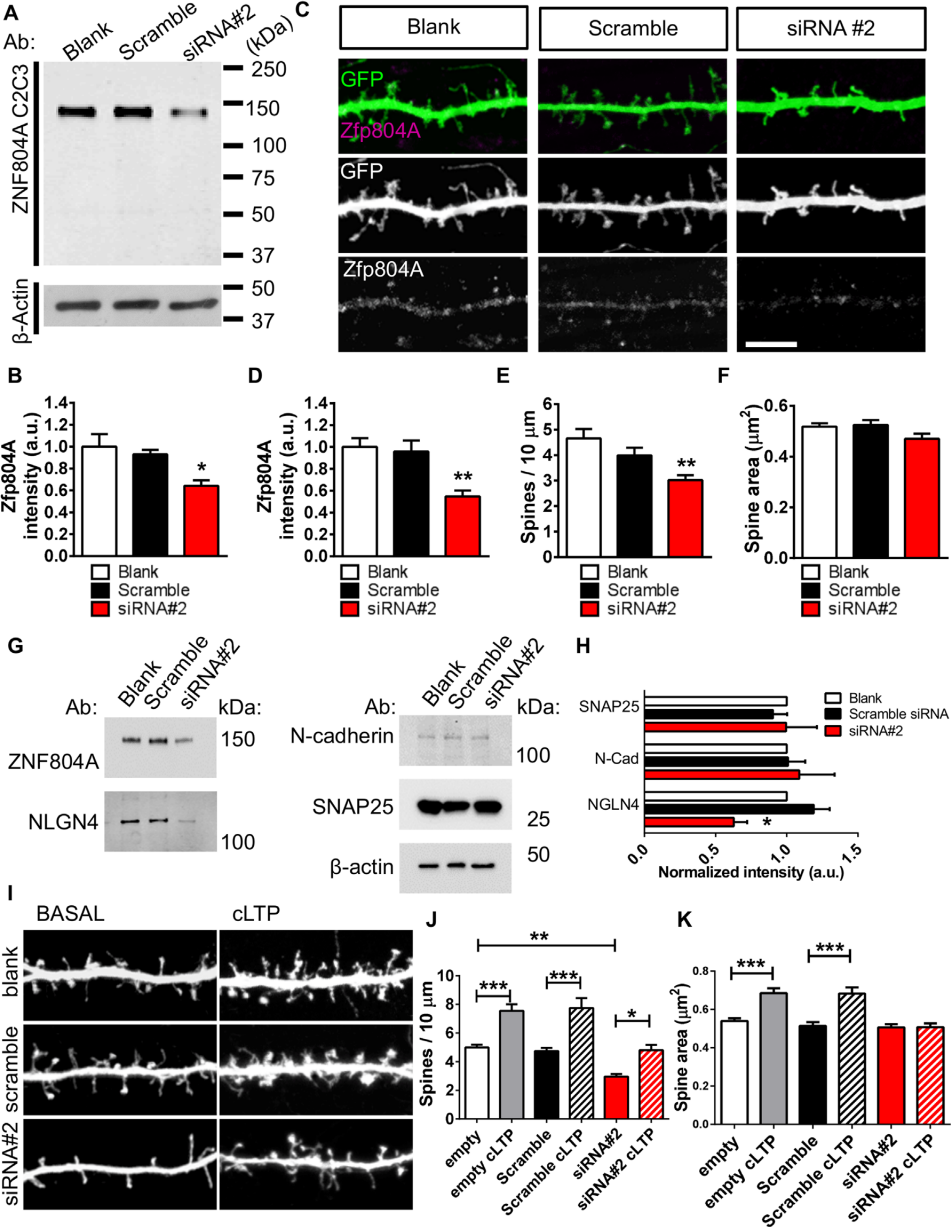
Small interfering RNA (siRNA)-mediated knockdown of zinc finger binding protein 804A (Zfp804A) in rat primary cortical neurons. **(A)** Representative Western blot of Zfp804A in 26-day in vitro (DIV26) primary rat cortical neurons after a 5-day treatment with no transfection (blank), control (scramble), or ZNF804A-targeting siRNA. **(B)** Quantification of the major protein band found at ~135 kDa, roughly equal to the predicted molecular mass of Zfp804A, revealed a significant reduction in staining intensity compared with blank and control conditions (*n* = 3 independent experiments). **(C)** Representative confocal images of dendrites and dendritic spines after siRNA treatment of neurons as in panel A. **(D)** Quantification of Zfp804A staining intensity after 5-day siRNA treatment. Zfp804A was significantly reduced in neurons treated with siRNA 2 in comparison with blank and control conditions (*n* = 3 independent experiments and at least 12 cells per condition). **(E)** Quantification of spine density after siRNA treatment (*n* = 3 independent experiments and at least 12 cells per condition). **(F)** Spine area was not found to be significantly altered in neurons treated with siRNA 2 relative to the two control conditions (*n* = 3 independent experiments and at least 12 cells per condition). **(G)** Western blot analysis of cell lysates of blank, control, and siRNA-treated neurons. **(H)** Quantification of panel F reveals that neuroligin-4 (NLGN4), but not N-cadherin or synaptosomal-associated protein 25 (SNAP25), is significantly reduced in Zfp804A knockdown cells (*n* = 3 independent experiments). **(I)** Representative confocal images of dendritic spines from neurons treated as in panel A and after chemical long-term potentiation (cLTP) stimulation (*n* = 3 independent experiments and 13–14 cells per condition). **(J)** Quantification of spine density in siRNA-treated cells after cLTP revealed that linear density was increased in all conditions; in Zfp804A cells, cLTP increased spine density back to basal levels. **(K)** Quantification of spine size (area) after cLTP revealed that loss of Zfp804A impaired the ability of cells to undergo structural plasticity (*n* = 3 independent experiments and 13–14 cells per condition). Scale bar = 5 µm. Ab, antibody; a.u., arbitrary unit; GFP, green fluorescent protein; N-Cad, N-cadherin. *p < .05, **p < .01, ***p < .001.

Our present data indicate that a subpopulation of Zfp804A localizes to synapses, suggesting a potential role in the maturation, or maintenance, of dendritic spines. Therefore, we knocked down Zfp804a using siRNA 2, predicted to also target Zfp804A; siRNA 2 reduced Zfp804A protein expression by ~35% (Figure 5A). A similar level of Zfp804A knockdown was observed by immunocytochemical analysis (Figure 5, Figure 5). Neurons treated with siRNA 2 for 5 days had ~35% fewer spines than neurons expressing no siRNA or scramble siRNA (Figure 5D). Interestingly, when we examined spine morphology, no difference was seen in spine area, length, or breadth, indicating that the reduction in linear density was due to a loss of spines of no particular morphology (Figure 5E and Supplemental Figure S10A–C). Because loss of ZNF804A in young human neurons correlated with a reduced NLGN4 expression, a protein known to regulate synapse density (21, 22), we examined whether this molecule was also affected by loss of Zfp804A expression. As predicted, NLGN4 was reduced in Zfp804A knockdown neurons, whereas no changes in the adhesion protein N-cadherin or the synaptic protein synaptosomal-associated protein 25 expression were observed (Figure 5, Figure 5).

Because altering Zfp804A expression influenced spine density, we reasoned that loss of Zfp804A may affect activity-dependent spine plasticity. Therefore, we used a well-characterized chemical long-term potentiation (cLTP) protocol shown to cause a rapid increase in spine area and density via activation of NMDA receptors (31, 39). Application of cLTP on control cells or neurons expressing scramble siRNA resulted in a significant increase in density (~33%) and spine area (~24%) (Figure 5, Figure 5). However, in Zfp804A knockdown neurons, no increase in spine area was observed after cLTP treatment, although spine density increased (~35%) to a level similar to unstimulated (basal) control levels (Figure 5, Figure 5). Collectively, these experiments demonstrate that Zfp804a is required for spine maintenance and that loss of the protein impairs the ability of neurons to respond fully to activity-dependent simulations.

## Discussion

Variants in *ZNF804A* are associated with various neuropsychiatric conditions (1, 2, 3, 4, 5, 6), and emerging evidence suggests that synaptic pathology may be a key cellular substrate contributing to the pathophysiology of these disorders (30). Whereas functional studies suggest that ZNF804A controls the expression of genes involved in cell adhesion, neurite outgrowth, and synaptic transmission (15, 19), little is known about the cellular function of this susceptibility gene. Here, we present evidence that ZNF804A/Zfp804a localizes to dendrites and synapses, in both neurons derived from either hNPC or hiPSC sources and mature rat cortical neurons. siRNA-mediated loss of ZNF804A further reveals a role in establishing early neuronal morphology, an effect potentially mediated by the ability of ZNF804A to regulate the expression of the adhesion protein NLGN4, a protein implicated in ASDs (21, 22). In mature neurons, super-resolution imaging revealed Zfp804A nanodomains at perisynaptic locations within dendritic spines, where it partially colocalizes with synaptic proteins. Critically, siRNA-mediated loss of Zfp804A resulted in a reduction in spine linear density and impaired activity-dependent structural plasticity. Collectively, these data suggest that ZNF804A/Zfp804A is involved in early neurite outgrowth and also plays a role in regulating spine maintenance and the ability of neurons to respond to activity-dependent stimuli. Although some of these cellular effects may be governed by the ability of ZNF804A/Zfp804A to regulate NLGN4 expression, ZNF804A/Zfp804a may also play a functional role at synapses and alter the expression of other synaptic proteins. Importantly, because risk variants in *ZNF804A* appear to alter its developmental expression (5, 12, 14), our data provide an insight into how this could contribute to the pathophysiology of psychotic and neurodevelopmental disorders.

ZNF804A has been posited as a putative transcription factor owing to the presence of a C2H2 zinc-finger domain, a motif that facilitates binding to specific DNA sequences (10, 11), and based on functional gene expression studies (15, 17, 19). Consistent with this role, Zfp804a has been reported to localize to the nucleus of rat progenitor cells (17). However, in silico analysis of ZNF804A has suggested that it may not act as a classical zinc-finger protein because it only contains a single C2H2 motif, in contrast to other members of the zinc-finger family that typically possess multiple zinc-finger motifs (10). Our data indicate that ZNF804A is highly present within the nucleus in undifferentiated hNPCs and throughout the cell. In differentiated neurons, a higher proportion of ZNF804A is found in the somatic compartment. This pattern of distribution has also been reported in developing rat neurons (18). Thus, ZNF804A appears to be enriched within the nuclear compartment of undifferentiated hNPCs, whereas in neurons it is distributed along dendrites, axons, and synapses.

Interestingly, our data suggest that there is a change in the cellular distribution of ZNF804A during neuralization, with more protein located in the nucleus of hNPCs and in extranuclear subcompartments in neurons. The change in distribution could be an indication of changes in isoform expression. Quantitative polymerase chain reaction analysis indicates that although *ZNF804A*^*E3/E4*^ expression is not altered during early neuralization, overall *ZNF804A*^*exon4*^ transcripts were reduced. The change in distribution could also reflect a change in the function of the protein. Indeed, the presence of ZNF804A along neurites/dendrites is consistent with our functional data revealing a role in establishing early neuronal morphology. However, it is currently unclear whether the different ZNF804A isoforms display different localizations or function differently. The recently discovered disease-associated transcript *ZNF804A*^*E3/E4*^ (12) lacks the C2H2-domain present in the full-length protein and thus has no obvious ability to bind DNA or act as a transcription factor. Therefore, to fully understand how this protein may contribute to the pathophysiology of psychiatric disorders it is critical to fully understand the distribution of all isoforms of ZNF804A and, furthermore, the functional roles they play.

In developing human neurons, ZNF804A partially colocalized with both PSD-95 and GluN1. Because these neurons can form immature synaptic connections (19), it is possible that ZNF804A is functionally present at putative synapses. Interestingly, ZNF804A knockdown attenuated neuritogenesis; concurrently, these cells display reduced expression of NLGN4. *NLGN4X* expression has recently been shown to be downregulated in ZNF804A knockdown hiPSCs (16), although it is not clear whether this is due to ZNF804A directly regulating *NLGN4X* expression. Although NLGN4 has been implicated in neuritogenesis (23), no direct evidence has been provided demonstrating a role in neurite formation. We show that exogenous-overexpression of NGLN4 rescues aberrant neurite outgrowth observed in ZNF804A knockdown neurons. Whether this indicates that the effects of ZNF804A on neurite outgrowth are dependent on the direct or indirect regulation of NLGN4 expression will need to be established in future studies.

In mature neurons, Zfp804A was predominately located along dendrites and within a subset of dendritic spines; it was also detected in axon terminals. Super-resolution imaging further revealed that Zfp804A could be found at the base of and within the head of spines. Within spine heads, Zfp804A appears to form nanodomains, which surround and integrate within the PSD. PSD-95 and GluN1 partially surrounded Zfp804A puncta, supporting an enrichment at perisynaptic regions, and a potential participation in a larger protein complex with synaptic proteins. The presence of ZNF804A at the base of dendritic spines is also potentially interesting when taken together with previous work showing that this subsynaptic compartment is a key region where regulation of messenger RNA occurs (40) and that C2H2 domains can mediate protein-RNA interactions (11, 41). We also find that loss of Zfp804A resulting in a reduction of dendritic spine density also occurred concurrently with a reduction in NLGN4 expression, which has also been implicated in the regulation of synapse density (21, 22). However, it is not yet clear whether Zfp804A also plays a functional role locally at synapses. Interestingly, our data also indicate that Zfp804A knockdown impairs rapid structural changes induced by cLTP, specifically in the enlargement of spine size. Although this effect could be driven by reduced expression of NLGN4 or other proteins, note that Zfp804A preferentially localized to small spines. It is these spines that readily undergo enlargement after cLTP (31); therefore, it is possible that Zfp804A functions locally at synapses in addition to regulating gene expression. Understanding whether Zfp804a contributes to local signaling at synapses, in addition to regulation of gene transcription, is crucial in further understanding the role of ZNF804A/Zfp804a in physiological and pathological conditions. It should be noted that although our data demonstrate a functional role for ZNF804A/Zfp804A in controlling neuronal and synaptic function based on siRNA-based approaches, interpretation of the distribution of the protein should be noted with caution. From our antibody characterization studies, the C2C3 antibody shows some level of nonspecificity by Western blot (Supplemental Figures S1–S5), although overexpression and knockdown studies indicate that C2C3 is specific for ZNF804A in immunocytochemical analysis (Supplemental Figures S2–S5). However, a true validation of antibody specificity can only be demonstrated using tissue lacking all isoforms. Nevertheless, our data examining the extracellular distribution are consistent with previous reports (12, 18) and critically with our overexpression/knockdown experiments and functional studies.

In conclusion, we find ZNF804A/Zfp804A to be expressed in discrete structures along the dendrites and at synapses and to have a functional role in regulating neuronal and synaptic morphology. Interestingly, the nanoscopic distribution of ZNF804A/Zfp804A in spines indicates a potential functional role in influencing local signaling at synapses. SCZ, BP, and ASDs have all been shown to display overlapping cellular deficits, including reduced dendritic arborization and spine density (30). In addition, increasing evidence suggests that risk factors implicated in these disorders converge onto a common biological function: control of neuronal and synaptic structure (30, 42). It is therefore possible that dysregulation of ZNF804A in these disorders could also contribute to altered neuronal and synaptic structures seen in these disorders (30). Our study further highlights how understanding the cellular function of risk proteins may reveal novel functions, but it may also identify how multiple psychiatric disorders may share genetic risk factors and biological mechanisms.
