# Supplementary material for: Psychosis Risk Candidate ZNF804A Localizes to Synapses and Regulates Neurite Formation and Dendritic Spine Structure
Source: Biol Psychiatry. 2017 Jul 1;82(1):49–61. doi: 10.1016/j.biopsych.2016.08.038 (PMC5482321; doi:10.1016/j.biopsych.2016.08.038)
Supplement: Supplementary file 1 — Supplementary material [file mmc1.pdf]

Psychosis Risk Candidate ZNF804A Localizes to Synapses and Regulates Neurite Formation and Dendritic Spine Structure

Supplemental Information

Supplemental Methods and Materials

Reagents

The ZNF804A (C2C3; GTX121178) rabbit polyclonal antibody was from Genetex; ZNF804A (D-14; sc-241170) goat polyclonal antibody was from Santa Cruz Biotechnology; and the ZNF804A (10F8.2; MABN706) mouse monoclonal antibody was from Millipore. GFP (ab13972), MAP2 (ab5392) and βIII tubulin (ab107216) chicken polyclonal, TBR1 (ab31940) rabbit polyclonal and neuroligin-4 (S98-7; ab18631) mouse monoclonal antibodies were from Abcam; PSD-95 (clone K28/43; 75-028), GluA2 (clone L21/32; 75-002) and GluN1 (clone N308/48; 75-272) mouse monoclonal antibodies were from NeuroMab. Vesicular glutamate transporter 1 (VGlut1; MAB5502), Nestin (MAB5326) mouse monoclonal, and Sox 2 (AB5603) rabbit polyclonal antibodies were from Merck Millipore; myc (9E10 clone; M4439) and N-cadherin (GC4, C3865) mouse monoclonal antibody was from Sigma.

The peGFP-C3 plasmid was from Clontech; GFP-ZNF804A (human) and myc-ZNF804A (human) plasmids were purchased from Origene; HA-NLGN4 (human) was a kind gift from Prof. Peter Scheiffele (University of Basel).

Cell Culture

Use of the CTX0E16 human neural progenitor cell (hNPC) line was kindly granted by ReNeuron Group plc. (Guildford, UK) under a Material Transfer Agreement.

Derivation of this conditionally immortalized hNPC line has been described previously (1, 2). CTX0E16 cells were maintained in a proliferative state by culturing in DMEM:F12 (Sigma) supplemented with 0.03% human serum albumin (PAA), 100 $\mu g ml^{-1}$ apo-transferrin (Scipac), 16.2 $\mu g ml^{-1}$ putrescine (Sigma), 5 $\mu g ml^{-1}$ human insulin (Sigma), 60 $ng ml^{-1}$ progesterone (Sigma), 2 mM L-glutamine (Sigma) and 40 $ng ml^{-1}$ sodium selenite (Sigma), 10 $ng ml^{-1}$ human FGF_2, 20 $ng ml^{-1}$ human EGF (PeproTech) and 100 nM 4-hydroxy-tamoxifen (4-OHT) (Sigma). Neuralization of CTX0E16 cells was achieved by replacing DMEM:F12 medium with Neurobasal Medium supplemented with B27 serum-free supplement (Life Technologies), 0.03% human serum albumin, 100 $\mu g ml^{-1}$ apo-transferrin, 16.2 $\mu g ml^{-1}$ putrescine, 5 $\mu g ml^{-1}$ human insulin, 60 $ng ml^{-1}$ progesterone, 2 mM L-glutamine, and 40 $ng ml^{-1}$ sodium selenite. Cells were grown on poly-D-lysine (0.2 mg/ml, Sigma) and laminin (1 $\mu g cm^{-2}$) coated glass coverslips (No. 1.5) until desired age (days differentiated; DD).

Dissociated cultures of primary cortical neurons were prepared from E18 Sprague-Dawley rat embryos and cultured as described previously (3). Neurons were plated onto glass coverslips (No. 1.5) coated with poly-D-lysine (0.2 mg/ml, Sigma), in feeding media: Neurobasal + B27 supplement + penicillin/streptomycin + 0.5 mM L-glutamine. Neuron cultures were maintained in presence of 200 μM D,L-amino-phosphonovalerate (D,L-APV, ab120004, Abcam) beginning on DIV 4. Cortical neurons were transfected at DIV 23 with a GFP plasmid using Lipofectamine 2000 (Life Technologies) (3); transfections were allowed to proceed for 2 days (DIV 25) before cells being fixed and processed for immunocytochemistry.

Human Induced Pluripotent Stem Cells (hiPSCs)

HiPSC lines were generated from primary keratinocytes as previously described (4). Briefly, 1 x 10^5 primary hair root keratinocytes were transduced with Sendai virus expressing OCT4, SOX2, KLF4 and C-MYC (kind gift of M. Nakanishi, AIST Japan) (5, 6). Transduced keratinocytes were plated onto an irradiated MEF feeder layer (Millipore) in supplemented Epilife medium for ten days before switching to 'hES media' consisting of KO-DMEM/F12 supplemented with 20% Knock-out serum replacement, Non-essential amino acids, Glutamax, b-mercaptoethanol (all from Life Technologies) and bFGF (10ng/ml; Peprotech). After a further two weeks, reprogrammed colonies were picked straight into E8 media (Life Technologies) on Geltrex coated plasticware. HiPSCs were validated by genome-wide expression profiling using Illumina Beadchip v4 and the bioinformatics tool 'Pluritest' (7), embryoid body formation and tri-lineage differentiation potential, ICC markers of pluripotency (4) including Nanog, OCT4, SSEA4 and TRA1-81, Alkaline phosphatase expression (Millipore), and for genomic stability by G-banded karyotyping (4).

HiPSCs were routinely maintained in E8 media on geltrex coated plasticware (ThermoFisher). Neuronal differentiation of hiPSCs was by achieved replacing E8 medium on confluent (>95%) hiPSCs with neuralization medium: 1:1 mixture of N2- and B27-containing medium supplemented with 5 µg ml^{-1} insulin, 1 mM l-glutamine, 100 µm non-essential amino acids, and 100 µM 2-mercaptoethanol. At this stage, neuralization medium was further supplemented with 1 µm Dorsomorphin (Sigma), and 10 µm SB431542 (Cambridge Bioscience) to inhibit TGFβ signaling during neural induction (8, 9). Cells were maintained in this medium until the appearance of neuroepithelial cells (day 8) (**Supplemental Figure 3A**), at which time cells were

passaged using Accutase (Sigma) and maintained in neuralization medium. Neuroepithelial cells were grown and passaged 3 times, until neural rosettes (neural progenitor cells (NPCs); ~day 17) formed (**Supplementary Figure 3B**). NPCs were passaged a further 2 times before terminal differentiation of hiPSCs into hiPSC-neurons (day 23); this was achieved by plating NPCs (day 23) onto poly-D-lysine (0.2 mg/ml) and laminin (1 µgcm^{-2}) coated glass coverslips (No. 1.5), and culturing with medium containing Neurobasal + B27 supplement. After 2 days (day 25) medium was supplemented with 10 µM DAPT for 5 days (day 30) to block NOTCH signaling; neurons were grown in Neurobasal + B27 medium until day 35 when they were used for experimentation.

Primary Neuronal Culture

Mixed sex cortical neuronal cultures were prepared from Sprague-Dawley rat E18 embryos as described previously (10). Animals were habituated for 3 days before experimental procedures, which were carried out in accordance with the Home Office Animals (Scientific procedures) Act, United Kingdom, 1986. Cells were plated onto 18 mm glass coverslips (No 1.5; 0117580, Marienfeld-Superior GmbH & Co.), coated with poly-D-lysine (0.2mg/ml, Sigma), at a density of 3x10^{5} cells/well equal to 857/mm^{2}. Neurons were cultured in feeding media: neurobasal medium (21103049) supplemented with 2% B27 (17504044), 0.5 mM glutamine (25030024) and 1% penicillin:streptomycin (15070063) (all reagents from Life technologies). Neuron cultures were maintained in presence of 200 µM D,L-APV beginning on DIV (days *in vitro*) 4 in order to maintain neuronal health for long-term culturing and to reduce cell death due to excessive Ca^{2+} cytotoxicity via over-active NMDA receptors (10). Half media changes were performed twice weekly until the desired age (DIV 23-25). The

primary cortical neurons were transfected with eGFP at DIV 23 for 2 days, using Lipofectamine 2000 (11668027, Life Technologies) (10). Briefly, 4 µg of plasmid DNA was mixed with Lipofectamine 2000, added dropwise to cells, incubated for 4-12 hours, before being replaced with fresh feeding media. Transfections were allowed to proceed for 2 days, which results in approximately 10% transfection efficacy (3, 10). For experiments involving the chemical induction of LTP (cLTP), rat primary cortical neuron cultures were plated and cultured until DIV25, with siRNA transfections occurring on DIV20 and eGFP transfections carried out on DIV23. At DIV25, transfected cultures were pre-treated with ACSF (in mM: 125 NaCl, 2.5 KCl, 26.2 $NaHCO_3$, 1 NaH_2PO_4, 11 glucose, 5 Hepes, 2.5 $CaCl_2$ and 1.25 $MgCl_2$) supplemented with D,L-APV for 60 minutes. Subsequently, cells were treated with ACSF with APV (basal) or ACSF supplemented with 10 µM glycine, 100mM picrotoxin and 1 µM strychnine, as the cLTP inducing stimuli, for 30 minutes prior to fixation (3).

Halo-ZNF804A Stably Expressing SH-SY5Y Cell Line

A vector containing full length human ZNF804A fused with the halotag sequence (Promega, UK) at the N-terminal was purchased from GeneCopoeia. To generate a stably expressing cell line, approximately 1 million SH-SY5Y cells were electroporated with 5 µg of plasmid using the Lonza nucelofector. 48 hours after transfection, growth media was replaced with selection media (growth media containing 800 µg/ml G418). Cells were expanded for 2 weeks to produce a polyclonal population of stable cells. Cells were further maintained in growth media containing 400ug/ml G418 until experimentation. Observation of halo-ZNF804A in live and fixed cells was performed by incubating cells with 1 µM of the

HaloTag^{R}TMRDirect^{TM} ligand (Promega, UK) for 15 minutes before imaging of live cells, fixation, or processing for Western Blotting.

siRNA Silencing of ZNF804A and Zfp804A

CTX0E16 cells, hiPSC-derived NPCs and rat primary cortical neurons were transfected using the N-TER Nanoparticle siRNA Transfection System. siRNA targeting full length ZNF804a mRNA sequence (Thermo Fisher: HSS150612 and HSS150613) **(Supplemental Table S1)** were used along with Stealth RNAi siRNA negative control med GC duplex (Invitrogen: 465372), which has no significant sequence similarity to human or rat gene sequences. Cells were transfected with the siRNAs using the N-Ter Nanoparticle siRNA Transfection System (Sigma: N2913) at DD0 (CTX0E16 cells), DD23 (hIPSC-derived cells) and DIV21 (rat primary cortical neurons) in accordance to manufacturer’s instructions, along with an N-Ter only blank control condition. In the case of CTX0E16 and hIPSC-derived neurons, cells were differentiated for 7 days, with a half media change occurring at day 4. For rescue experiments, CTX0E16-neurons were transfected with HA-NLGN4 at DD5 using Lipofectamine 2000 (LFA2K) (Life technologies: 11668027), and cells fixed at DD7. Cells were assessed for transfection efficiency in the fluorescent control condition the day after transfection, and were fixed or harvested at day 7 of differentiation. In the case of the rat primary cortical neurons, cells remained in the siRNA media for 5 days after which they were processed for western blotting. Rat neurons were transfected with peGFP-C3 at day 3 using LFA2K before being double fixed at day 5 and processed for immunocytochemistry. Transfection efficiency in all three cellular systems was assessed the day following siRNA transfection via live cell imaging using an inverted epifluorescence microscope with a 10x objective and

manually performing cell counts of the number of cells expressing the fluorescent oligonucleotide relative to the total number of cells. In each cellular system three independent experiments were carried out, with two technical replicates each and 3 fields of view taken per replicate. Based on the uptake of a red fluorescent oligonucleotide (BLOCK-iT), transfection efficiency was approximately 85% in CTX0E16 cells, and 63% across in hiPSCs.

RNA Preparation, cDNA Synthesis, RT-PCR and q-PCR

cDNA samples from proliferative and differentiated CTX0E16 cells and hiPSC-derived neurons were generated as described previously (11). To determine the expression of specific genes, primer sequences from previously established amplicons were designed to target all known RefSeq transcripts of genes of interest, sourced from the UCSC Genome Browser website (http://genome.ucsc.edu) **(Supplemental Table S2)**. Reactions were carried out in a total volume of 20 µl containing diluted cDNA, 1 X HOT FIREPol Blend Master Mix (Solis Biodyne) and primers at 200 nM, using a GS4 thermal cycler. Samples were separated and visualised by polyacrylamide gel electrophoresis.

For quantitative expression analysis, 20 µl cDNA samples from SuperScript III reactions were diluted with a further 100 µl of nuclease-free H_2O. Reactions were carried out in a total volume of 20 µl, containing diluted cDNA, 1× HOT FIREPol® EvaGreen® q-PCR Mix (Solis Biodyne, Tartu, Estonia) and primers at 200 nM, using an MJ Research Chromo 4 (Bio-Rad) and MJ Opticon Monitor analytic software (Bio-Rad). Triplicate q-PCR reactions were performed and averaged to measure each gene in each cDNA sample. The mean measures of target genes were normalized against a geometric mean determined from 3 internal control genes (RPL30, HPRT1

and RPL13A) for each cDNA sample to yield a relative target gene expression value for all samples using the Pfaffl method. The amplification efficiency of each set of primers was measured using a standard curve constructed by serial dilution of pooled cDNA from all assayed samples; only primer sets with efficiencies between 1.8 and 2.2 were used in order to ensure reproducibility of results. A Kruskall-Wallis test with posthoc Dunn's multiple comparisons test was performed on the normalized qPCR data for relative expression of the full length ZNF804A transcript using Graphpad Prism.

Western Blotting

Western Blotting was performed using 10-30 µg protein loaded on 4-20% gradient acrylamide gels (Bio-Rad Laboratories: 4561094) for both CTX0E16 and rat neurons treated with siRNAs; 25 µg protein was loaded for rat neuron crude synaptosomes. Western blots were blocked in TBS with 0.1% Tween-20 with 5% bovine serum albumin (BSA). The following antibodies were used: ZNF804A (C2C3) rabbit polyclonal (Genetex: GTX121178) at 1:250; ZNF804A rabbit polyclonal (Biorbyt: orb1856) at 1:100; ZNF804A (D-14) goat polyclonal (Santa Cruz: sc-241170) at 1:100; PSD-95 (clone K28/43) mouse monoclonal (Neuromab: 73-028) at 1:1000; beta-actin loading control (BA3R) mouse monoclonal (Fisher Scientific: 11355703) at 1:2000. All blots were incubated with a secondary antibody conjugated to horseradish peroxidase and developed using the chemiluminescence method with Luminata Crescendo HRP (Fisher Scientific: 11790644). The optical density of the bands of ZNF804A was measured using ImageJ and normalized to the optical densities of the respective β-actin bands. The values for each condition were then

normalized to control condition, and the mean and standard errors were calculated for all biological replicates.

Immunocytochemistry (ICC) and Microscopy

To assess the efficiency of ZNF804A knockdown using siRNA transfection in both the CTX0E16 cells and hiPSC-neurons, representative z-stacks of triple-stained cultures were taken using a Leica SP5 confocal microscope with a 40x objective (N.A. 1.4) with at least 5 frames per z-stack at steps of 0.5 µm. Images were z-projected and background subtracted in Metamorph. Regions were traced around the perinuclear cytoplasm and initial 10 µm of primary neurites of hiPSC-neurons as determined by MAP2 staining in ImageJ (due to these regions being the site of the greatest ZNF804A staining intensity); these were transferred to the ZNF804A staining channel and the average staining intensity was measured in each. For CTX0E16 cultures regions for analysis of ZNF804A expression were derived from binarized images of the DAPI-stained cell nuclei. Average intensity values were averaged within a single technical replicate, and values for technical replicates were averaged within each biological replicate. CTX0E16 cell line biological replicates were defined as cultures derived from different passages. Technical replicates were defined as separate cultures derived from a single passage. HiPSC biological replicates were defined as individual lines (M1, M2, and M3). Technical replicates were defined as separate cultures from a single passage of a given hiPSC line. 3-5 Images were taken per coverslip, and 1-2 technical replicates per biological replicate. 3 biological replicates in total were performed for experiments with the CTX0E16 line. Cultures derived from hiPSC lines were sampled as above, but with 2 biological replicates per cell line and 2 hiPSC lines used in total.

To determine the relative subcellular expression of ZNF804A using immunocytochemistry, DD0 and DD28 CTX0E16 cultures were fixed, immunostained for ZNF804A and βIII-Tubulin and imaged using confocal microscopy with a 63x objective as described previously. 3-5 fields of view (FOV) per technical replicate were imaged and 1-2 technical replicates per biological replicate. 3 biological replicates in total were performed. Regions of interest were traced around DAPI-positive cell nuclei as well as the cytoplasm of each cell used in analysis using ImageJ. These were then transferred to the ZNF804A staining channel and average staining intensities were measured for each region of interest. For each cell, relative expression of ZNF804A in both the nuclear and cytoplasmic regions was normalized to an overall expression value for the whole cell. These values were then averaged within each technical replicate and then within each biological replicate. Finally, overall averages for each subcellular compartment were calculated for both DD0 and DD28 CTX0E16 cells.

Structured Illumination Microscopy

The normal resolution of conventional confocal microscopy is limited to ~200 nm. Therefore, to improve the spatial resolution of our immunostaining data, we used structural illumination microscopy (SIM) (12). This form of microscopy gives the possibility of exceeding this diffraction limit; SIM increases the normal lateral resolution theoretically by a factor of two, and provides the same increase in axial direction (12).

SIM imaging was carried out on primary rat cortical neurons transfected with GFP and co-immunostained for ZNF804A (Genetex) and either PSD-95 or GluN1. Images were acquired with a Nikon N-SIM super-resolution microscope. Acquisition

was performed in a 3D SIM mode using the 100X 1.49 NA TIRF objective lens with 13-25 z-steps per stack at a step interval of 0.12 µm. Raw images were acquired on an Andor DU897 EMCCD camera at 512 x 512 pixels (62 nm pixel size in the image). Reconstructed images are displayed with 1024 x 1024 pixels (32 nm pixel size in the image). Image reconstructions were performed with Nikon N-SIM software. Final images, including z-projections and brightness and contrast adjustments were reconstructed in ImageJ and Metamorph.

Quantitative Analysis of Spine Morphologies

Confocal images of double-stained neurons were acquired with a Leica SP-5 confocal microscope using a 63x oil-immersion objective (Leica, N.A. 1.4) as a z-series. Two-dimensional maximum projection reconstructions of images were generated and morphometric analysis (spine number, area and breadth) was done using MetaMorph software (Universal Imaging Corporation) (10). Morphometric analysis was performed on spines from at least two dendrites (secondary or tertiary branches), totalling 100 µm, from each neuron. Linear density and total gray value of each synaptic protein cluster was measured automatically using MetaMorph (10). Cultures directly compared were stained simultaneously and imaged with the same acquisition parameters. For each condition, 9-16 neurons from at least 3 separate experiments were used. Experiments were carried out blind to condition and on sister cultures.

Neurite Outgrowth

In order to assess the impact of siRNA-mediated knockdown of ZNF804A on early neurite outgrowth on immature neurons derived from CTX0E16 and hiPSC lines,

representative images were taken of double-stained cultures using a Zeiss Axio Imager Z1, equipped with an AxioCam MR3 camera running AxioVision 4.7.1 imaging software (Carl Zeiss). These images were background subtracted in ImageJ and analyzed using a neurite outgrowth application in Metamorph. This application uses the nuclear staining channel image to mark potential cells and then traces neurites and marks neuronal cell body contours in the MAP2 (hiPSC-neurons) or βIII-Tubulin (CTX0E16-neurons) staining channel of the same image. The number of neurites per cell, total neurite length and average length of neurite per cell, number of neurite branch points per cell and cell soma area were recorded. Cells without neurites exceeding 5 µm or with cell somas exceeding 500 μm^2 in area were excluded from the analysis to exclude any non-neuronal cells in the FOV. During quantification the images were also assessed manually to ensure the neurite outgrowth application was tracing neurites accurately; any cells or images unable to be traced accurately by the application were removed from subsequent analysis. For rescue experiments, cells were imaged with a Lecia SP5 confocal, using a 40x (N.A. 1.4) objective, and neurite outgrowth measured using the NeuronJ plugin for ImageJ.

Values were averaged for cells from within a single technical replicate. Values for technical replicates were then averaged within each biological replicate for each experimental condition. 3-8 Images were taken per coverslip, and 1-2 technical replicates per biological replicate. 3 biological replicates were performed in total for cultures derived from the CTX0E16 line. Cultures derived from hiPSC lines were sampled as above, but with 2 biological replicates per cell line and 3 hiPSC lines used in total.

Statistical Analysis

For all experiments, 2-4 technical replicates from 3-4 independent experiments were used. For all graphs, bars represent means, and error bars as standard error mean (S.E.M). All statistical analysis was performed in GraphPad. To identify differences among conditional means, statistical analyses (Student's unpaired *t*-test, analysis of variance (ANOVA): for comparisons between multiple conditions the main effects and simple effects were probed by 1- or 2-way ANOVAs with Tukey correction for multiple comparisons.

Supplemental Results

Characterization of ZNF804A C2C3 Antibody in CTX0E16 Cell Line and Primary Rat Neurons

The C2C3 ZNF804A antibody was raised against a region within the C-terminal of human ZNF804A, and therefore should detect full length (FL) ZNF804A, as well as E3/E4 and variant b isoforms (**Supplemental Figure S1A**). RT-PCR and qPCR analysis of transcript expression indicates that ZNF804A$^{FL, E3/E4}$ and $^{variant\ b}$ isoforms are expressed in CTX0E16 cells (**Supplemental Figure S1B-C**). To evaluate the specificity of the C2C3 antibody, we first tested it by Western blotting using whole cell lysates from DD30 CTX0E16-neurons and DIV25 rat cortical neurons. The C2C3 antibody detected a major ~135 kDa protein band and minor protein bands at >100 kDa, 75 kDa, ~60 kDa and ~40 kDa in DD30 CTX0E16-neuron cell lysates (**Supplemental Figure S1D**). The ~135 kDa protein band is the mass predicted to be encoded by FL ZNF804A, and the >100 and 75 kDa bands are small enough to be encoded by the E3/E4 and variant b ZNF804A isoforms. It is of note that qPCR analysis only detects low levels of the E3/E4 transcript (**Supplemental Figure S1C**); similarly the protein band matching the predicted mass of this isoform is only observed at low levels by Western blotting (**Supplemental Figure S1D**). The ~135 kDa band could readily be seen in DD30 CTX0E16 cell lysate immunoprecipitated with the C2C3 antibody; again faint bands of ~100, 75, 60 and 40 kDa could also be detected (**Supplemental Figure S1D**); however, the 40 kDa protein could also be observed in control IgG immunoprecipitated cell lysate, indicating that it was due to non-specific binding (**Supplemental Figure S1D**). Preincubation of the C2C3 antibody with antigenic peptide prevented immunoprecipitation of the ~135, ~100 and 75 kDa proteins, demonstrating that binding to the protein was specific; a

possible band at 40 kDa could potentially be seen, but owing to signal from the IgG band, we were unable to confirm the presence of this protein in the pre-immune condition (**Supplemental Figure S1D**). Further work is required to uncover the identity of the minor proteins observed at ~100, 75 and 60 kDa. In whole cell lysate derived from DIV25 primary rat cortical neurons, a single major band could be observed at ~140 kDa (**Supplemental Figure S1E**); this mass is higher than the predicted weight of Zfp804A, but could be due to post-translational modifications. Immunoprecipitation with C2C3 as well as pre-immune and IgG control experiments further support that the ~140 kDa protein was specifically being bound to by the C2C3 antibody (**Supplemental Figure S1E**).

To confirm the specificity of the C2C3 antibody to detect full length ZNF804A, we transfected a myc-tagged ZNF804A construct into hEK293 cells, which has previously been shown to not express ZNF804A at high levels (13). Probing of Western blots with an anti-myc antibody revealed a major protein at ~135 kDa (**Supplemental Figure S1F**). Interestingly, additional bands could be detected at lower weights, which may be indicative of protein degradation. Probing of the same lysates with the C2C3 ZNF804A also detected proteins that corresponded to those detected by the myc antibody demonstrating that C2C3 specifically detects full length ZNF804A (**Supplemental Figure S1G**). Collectively, these data indicate that the C2C3 antibody specifically detects full length ZNF804A/Zfp804A, but that some minor non-specific band of low mass are also detected.

We next sought to validate the C2C3 antibody for ICC; DD0 CTX0E16-hNPCs were labelled with C2C3 or C2C3 pre-absorbed with its antigen. Cells labelled with the C2C3 antibody alone displayed punctate immunoreactivity within the nucleus and cytosol of hNPCs (**Supplemental Figure S2A**). Conversely, preincubation with

antigenic peptide almost completely blocked any immunoreactive signal (**Supplemental Figure S2A**); it is of note that some fluorescent signal could be detected however, it is currently unclear what the source of this signal may be. We once more took advantage of the low expression levels of ZNF804A in hEK293, and transfected these cells with a GFP-tagged human ZNF804A construct; cell were subsequently fixed and double immunostained with GFP and C2C3 antibodies. GFP-ZNF804A could be observed within the cell nucleus as well within the cytoplasm; the protein was also found to accumulate at the plasma membrane of the cells (**Supplemental Figure S2B**). Examination of the C2C3 channel revealed that it almost entirely overlapped with the GFP fluorescent signal. This can be readily seen in the colocalization image, which highlights where the GFP and C2C3 channels overlap (**Supplemental Figure S2B**).

To further investigate the specificity of the C2C3 antibody for ICC, we utilized 2 previously characterised siRNAs for ZNF804A (14). CTX0E16 cells were transfected with no siRNA (blank), scramble siRNA (control), siRNA#1 or siRNA#2 for the first 7 days of differentiation, before being processed for qPCR or ICC (**Supplemental Figures S3A-D**). Analysis of full length ZNF804A expression by qPCR revealed that both siRNA#1 and #2 reduced ZNF804A mRNA by ~40%. In parallel, a subset of cells were processed for ICC; cells were immunostained for βIII tubulin (morphological marker), the C2C3 ZNF804A antibody and the 10F8.2 ZNF804A antibody (Millipore). ZNF804A expression was assessed by measuring the fluorescent signal in the C2C3 and 10F8.2 channels in βIII tubulin positive cells. The immunoreactivity in the C2C3 channel was significantly reduced in siRNA#1 and #2 expressing cells as compared to blank or scramble siRNA conditions (**Supplemental Figure S3C**) indicating that C2C3 immunoreactivity was specific for full length

ZNF804A. Interestingly, the florescent signal generated by the 10F8.2 antibody did not differ between the conditions (**Supplemental Figure S3D**). Consistent with these results, probing of Western blotting of cell lysates taken from CTX0E16-neurons (DD7) transfected with no plasmid, scramble siRNA, siRNA#1 or siRNA#2, with the C2C3 antibody revealed that the major ~135 kDa protein band was significantly reduced in the siRNA#1 and #2 conditions thus, providing further evidence that the C2C3 antibody specifically detects full length ZNF804A in CTX0E16 neurons (**Figure 1A**). Previous studies have used the D-14 ZNF804A antibody (Santa Cruz) to examine the distribution of ZF804A in human and rat neurons (13, 15, 16). Thus, we probed Western blots of cell lysates of CTX0E16 neurons transfected with no plasmid, scramble siRNA, siRNA#1 or siRNA#2. Major bands could be observed at ~100 and ~80 kDa, whereas a faint band at ~135 kDa could be observed in addition to higher molecular weight products and several lower mass products. Interestingly, the bands seen at ~135, 100 and 80 kDa were reduced in the siRNA~1 and #2 conditions indicating that this antibody is specific for ZNF804A; however, the presence of additional protein bands suggests that this antibody may not be as specific as C2C3 (**Supplemental Figure S3D**). In a final set of control experiments, we generated a polyclonal population of SH-SY5Y cells stably expressing full length human ZNF804A with an N-terminally fused halo tag (halo-ZNF804A). This allowed us to observe the distribution of ZNF804A in a subpopulation of live and fixed cells, with the HaloTag^{R}TMRDirect^{TM} ligand (**Supplemental Figure S5**). In live cells, halo-ZNF804A could be seen in both nuclear and extranuclear compartments (**Supplemental Figure S5A**), similar to exogenously expressed GFP-ZNF804A and endogenous protein in hNPCs. A similar distribution was also observed in fixed cells, and C2C3 immunofluorescence could be seen to overlap with the halo signal;

however, non-transfected cells were also positive for ZNF804A, albeit at a lower level (**Supplemental Figure S5B**). We believe this to be endogenous expression of the ZNF804A protein in wildtype SH-SY5Y cells. Importantly, when we performed Western blotting on cell lysates from wildtype or halo-ZNF804A-expresing SH-SY5Y cells, a single band at >150 kDA was detected by the HaloTag^{R}TMRDirect^{TM} ligand, a size consistent with a fusion of the halo tag and full length ZNF804A (halo tag = 33 kDa, ZNF804A = 135 kDa, total predicted weight of fusion protein = 168 kDa; **Supplemental Figure S5C**). A protein band of the corresponding weight was also detected by the C2C3 antibody in the halo-ZNF804A condition, but not wildtype conditions (**Supplemental Figure S5C**). It should be noted that additional bands of ~140 kDa and lower could be detected in both wildtype and halo-ZNF804A conditions (**Supplemental Figure S5C**). It is likely that these bands represent the endogenous protein expressed by these cells; however, we cannot rule out the possibility that these are non-specific bands.

Taken together, these data indicate that the C2C3 antibody specifically identifies full length ZNF804A/Zfp804A by Western blotting. However, several additional bands are detected in control conditions, indicating that the antibody does exhibit some degree of non-specificity by Western blotting. Interestingly, our ICC data suggests that the C2C3 antibody is specific for ZNF804A/Zfp804a in multiple cells types. This includes, loss of immunofluorescent signal following knockdown with by 2 independent and previously validated siRNAs (14), and colocalization with exogenously or stable expressed tagged human ZNF804A constructs. Nevertheless, a further validation of this antibody will need to be performed using ZNF804A/Zfp804A knockout tissue, to ascertain the true specificity of this antibody in different experimental approaches.

Supplemental Table S1. Sequence of siRNAs against ZNF804A.

**siRNA ID**	**ZNF804A siRNA#**	**Sense sequence**
HSS150612	1	CCUAUAGCUGUAAUCCUCUAUGUUU
HSS150613	2	CCAGGAGUUUGACAAUCACAUUAAU

Supplemental Table S2. Primer sequences used in RT- and qPCR analysis.

	**Oligo sequence**	
	**Forward (5'-3')**	**Reverse (5'-3')**
**ZNF804A Exon 4**	GAT TTG TCC CCA GTG CTT GT	GCC TCT GGT GGA TGA AAA GA
**E3/E4 Variant**	CAA GCC AAA ATG CGA GAA AAT ATT	CCT TGT CGA GAG GTA AAC ACA ACA
**Variant C**	GAA TGA GGC AGC ATG CAG TA	TGG GAT CAA AGA CTG GGT TC
**RPL13A**	CCT GGA GGA GAA GAG GAA AGA GA	TTG AGG ACC TCT GTG TAT TTG TC
**RPL30**	ACA GCA TGC GGA AAA TAC TAC	AAA GGA AAA TTT TGC AGG TTT
**SDHA**	TGG GAA CAA GAG GGC ATC TG	CCA CCA CTG CAT CAA ATT CAT G
**B2M**	TAT CCA GCG TAC TCC AAA GA	GAC AAG TCT GAA TGC TCC AC
**UBC**	ATT TGG GTC GCG GTT CTT G	TGC CTT GAC ATT CTC GAT GGT
**GAPDH**	ATG GCA AGT TCA AAG GCA CAG TCA	TGG GGG CAT CAG CAG AAG G

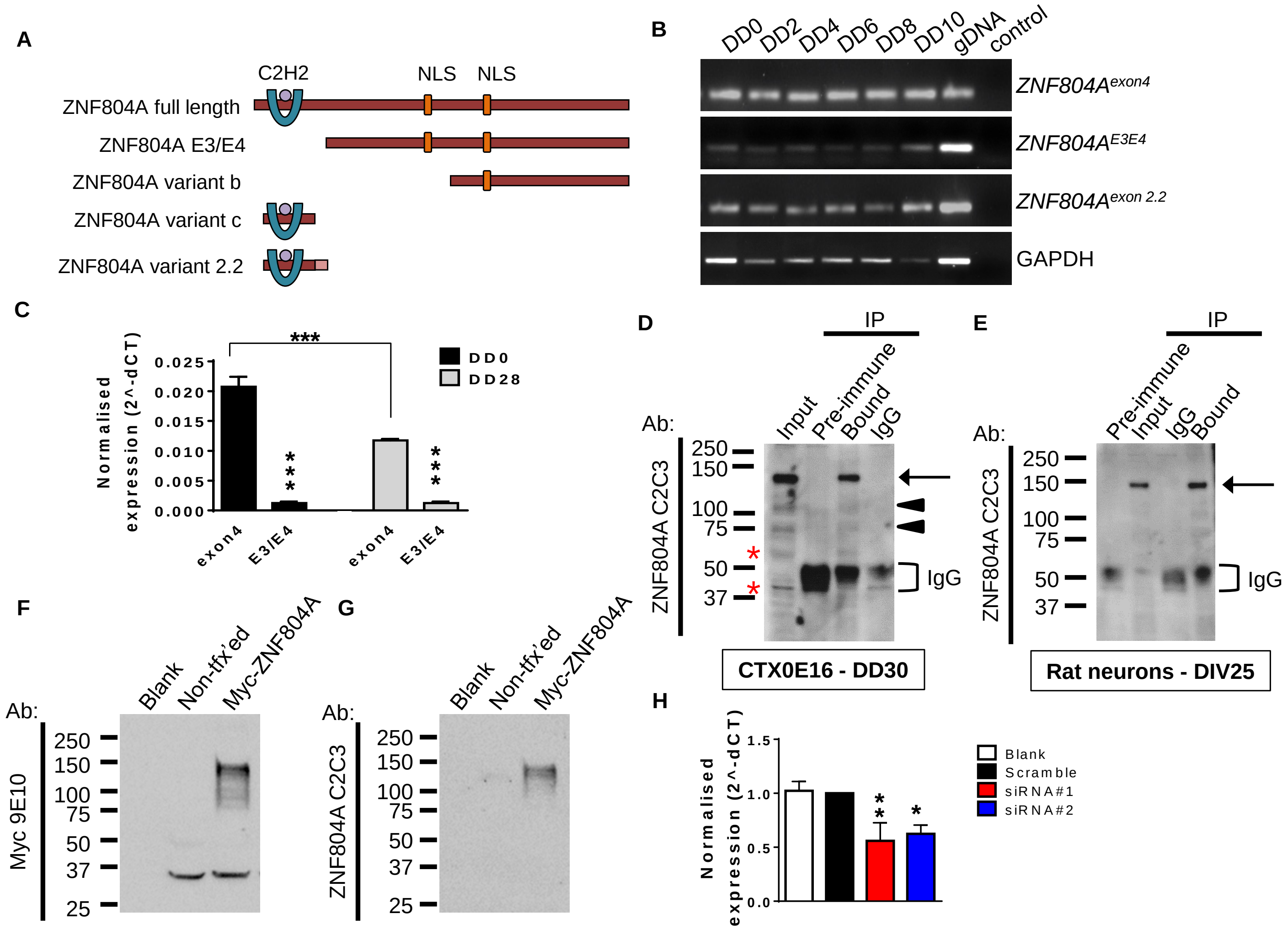

Supplemental Figure S1: Expression of *ZNF804A* transcripts in CTX0E16 cells and validation of ZNF804A C2C3 antibody in Western blotting. (A) Schematic of domain structure of predicted ZNF804A protein isoforms. **(B)** RT-PCR of mRNA for *ZNF804A*$^{exon4,\ E3/E4}$ and $^{exon\ 2.2}$ transcripts in proliferative and early-stage differentiated cells derived from the CTX0E16 cells. **(C)** QPCR of mRNA for *ZNF804A*^{exon4} and $^{E3/E4}$ transcripts in proliferative and early-stage differentiated cells derived from the CTX0E16 line (n = 3 independent experiments; ***p<0.001). **(D)** Western blot of DD30 CTX0E16 cell lysates immunoprecipitated with the C2C3 antibody, and probed using this antibody with or without pre-incubation with 10 µg of antigenic peptide. Black arrow denotes band corresponding to the predicted mass of the full length ZNF804A protein; black arrow heads denote minor bands potentially corresponding to other known or predicted ZNF804A protein isoforms. Red asterisks denote non-specific bands. **(E)** Western blot of DIV25 rat primary cortical neuron lysates immunoprecipitated with the C2C3 antibody and probed using this antibody with or without pre-incubation with 10 µg of antigenic peptide. Black arrow denotes band corresponding to the predicted mass of the full length Zfp804A protein. **(F)** Western blot of hEK293 cell lysates untransfected or transfected with a myc-tagged ZNF804A construct and probed with an anti-myc antibody; note the presence of additional

smaller bands potentially indicating degradation of the overexpressed protein. **(G)** Western blot shown in **(F)** but probed with the C2C3 anti-ZNF804A antibody; C2C3 specifically detects myc-ZNF804A. **(H)** QPCR of mRNA for *ZNF804A*^{exon4} transcripts following 7 day siRNA treatment in DD7 CTX0E16-neurons.

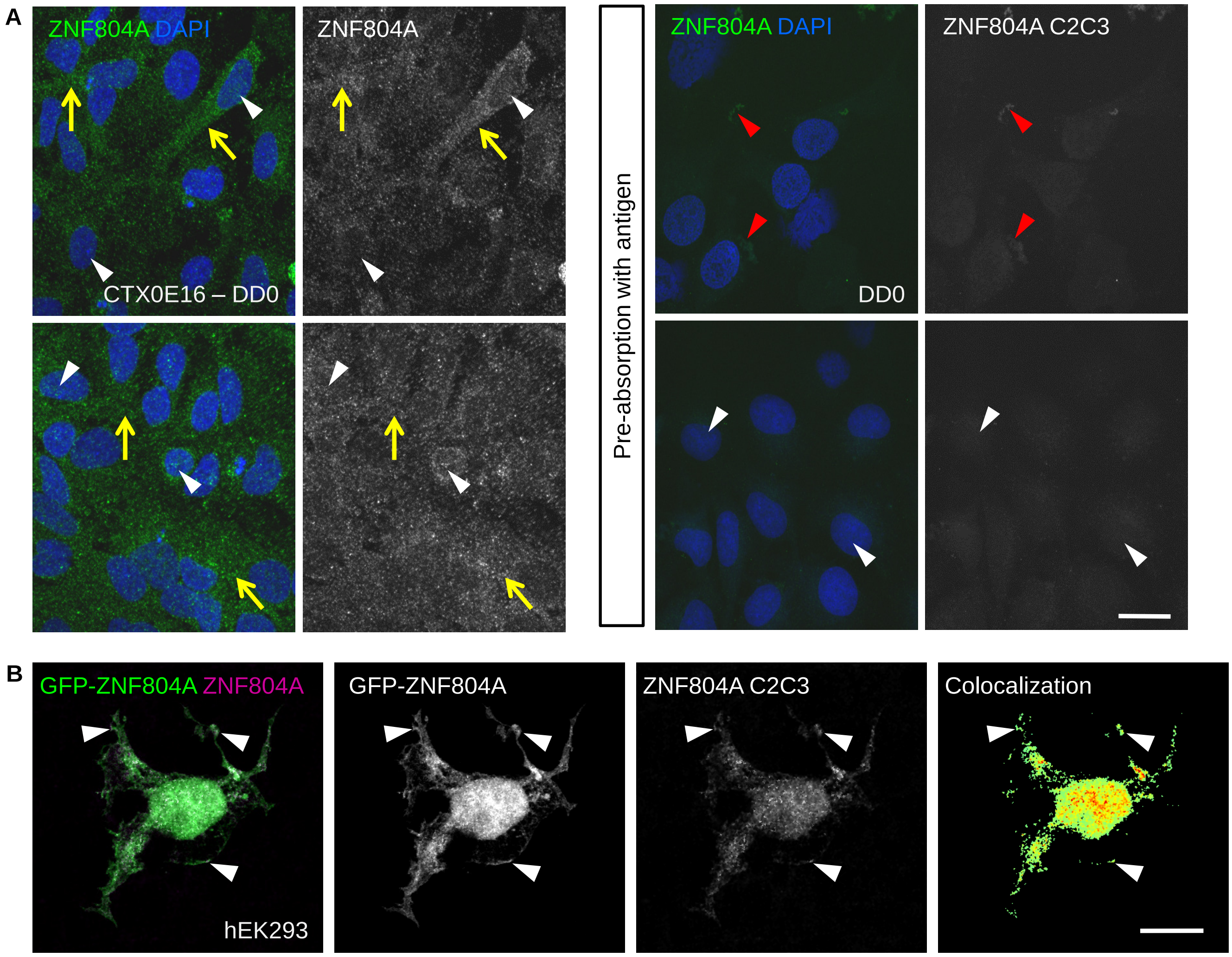

Supplemental Figure S2: Validation of the ZNF804A C2C3 antibody for ICC using peptide preabsorption and GFP-tagged ZNF804A construct overexpression. (A) Representative confocal imaging of DD0 CTX0E16 cells immunostained with the ZNF804A C2C3 antibody with or without pre-incubation 10 µg of antigenic peptide. Yellow arrows indicate immunoreactive staining within the cytoplasm of CTX0E16 cells while white arrowheads highlights staining within cell nuclei. Note the pre-absorption with antigen abolished the granular staining produced by the ZNF804A C2C3 antibody alone; red arrow heads indicates possible non-specific staining. **(B)** Confocal image of hEK293 cell transfected with a GFP-tagged ZNF804A construct and doubled immunostained with GFP and ZNF804A C2C3 antibodies. White arrowheads highlights ZNF804A expression within cellular processes, and indicates overlapping GFP and ZNF804A C2C3 immunostaining. Note the colocalization of GFP and ZNF804A C2C3 staining as shown in the final panel. Scale bar = 10 µm **(A + B)**.

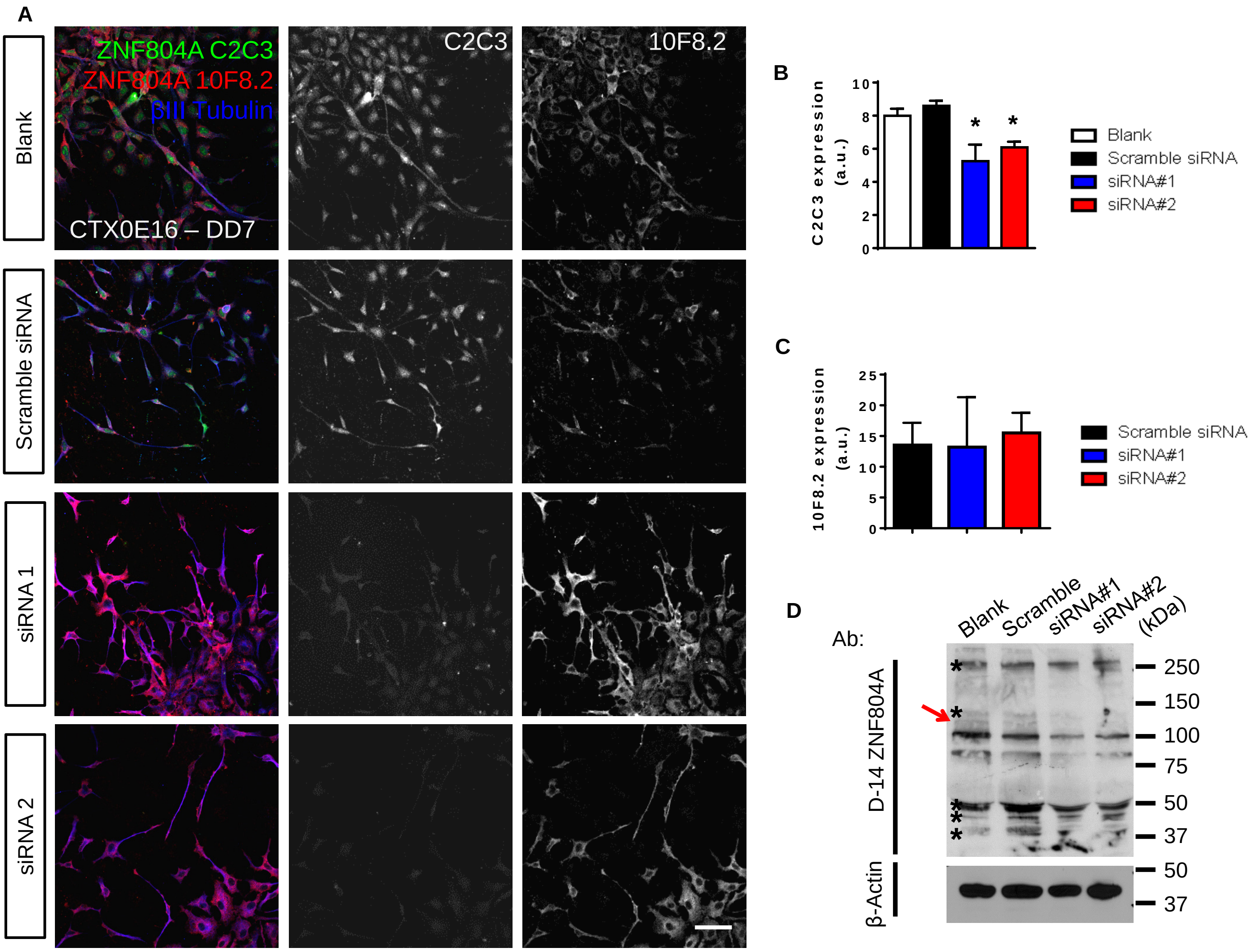

Supplemental Figure S3: siRNA treatment of CTX0E16 cultures reduces expression of ZNF804A. (A) Representative confocal images of DD7 CTX0E16 cultures treated with a blank control condition (no transfection), a scramble siRNA or one of two siRNAs targeting the full length *ZNF804A* transcript for 7 days and immunostained for ZNF804A with two independent antibodies (C2C3 and 10F8.2) in addition to βIII tubulin as a marker of neuronal cells. **(B + C)** Quantification of ZNF804A C2C3 and 10F8.2 staining as seen in **A**. SiRNA treatment of CTX0E16 cells significantly reduced C2C3 staining intensity in confocal images relative to control or scramble siRNA conditions; no change in 10F8.2 immunoreactivity was observed across all conditions. **(D)** Western blot of ZNF804A following 7 day siRNA treatment in DD7 CTX0E16 cells probed with the Santa Cruz D-14 anti-ZNF804A antibody. Red arrow denotes protein band corresponding to the predicted size of the full length ZNF804A protein, asterisks indicate non-specific bands. Scale bar = 20 µm.

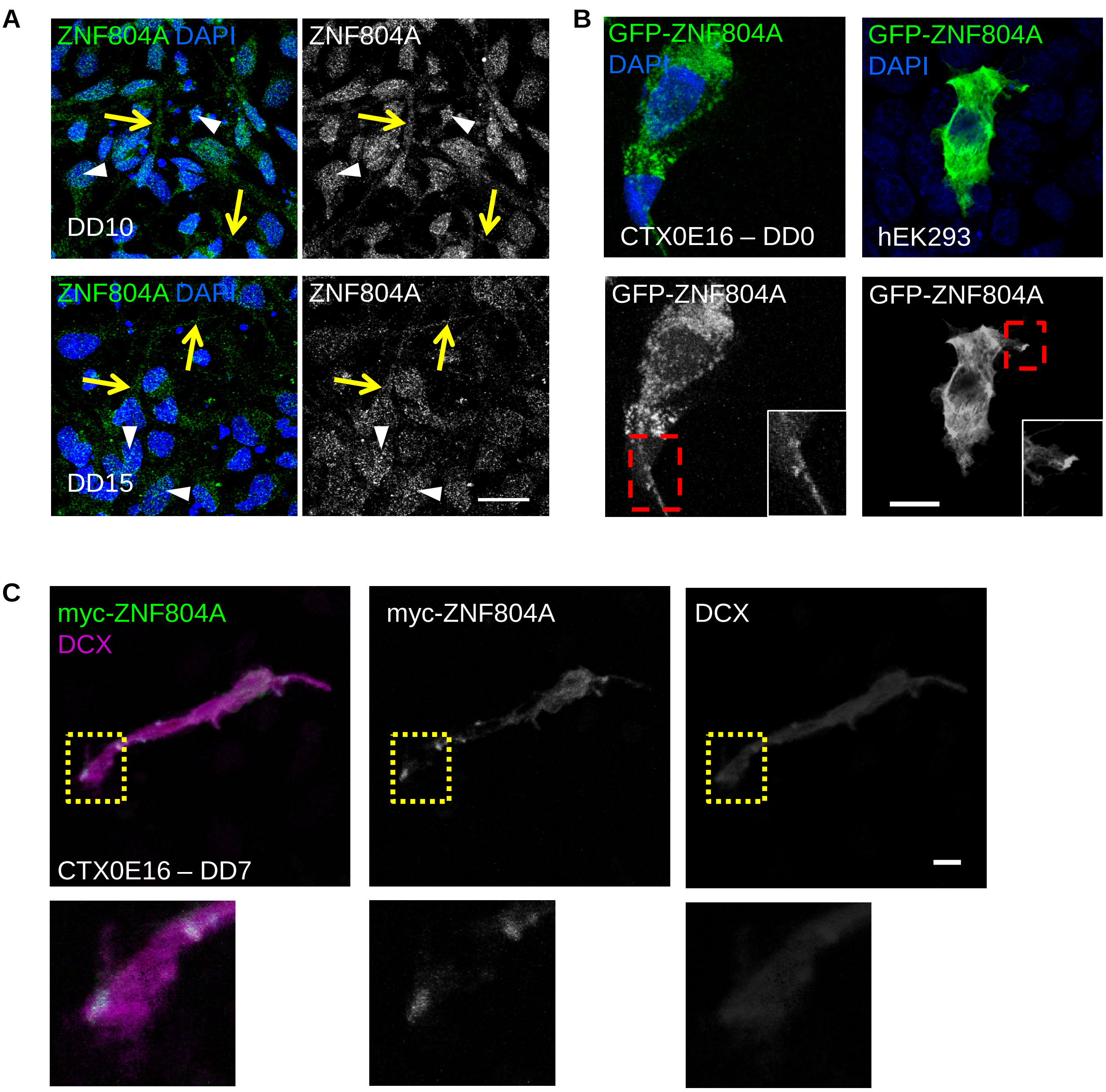

Supplemental Figure S4: Distribution of endogenous and exogenous ZNF804A in young CTX0E16 and hEK293 cells. (A) Differentiated day (DD) 10 or 15 CTX0E16 neurons, immunostained for ZNF804A using C2C3 antibody. In both DD10 and 15 neurons, ZNF804A was found within the nucleus (white arrow heads) and in the cytosol and neurites (yellow arrows). **(B)** Localization of exogenously expressed GFP-ZNF804A in DD0 CTX0E16 cells and hEK293 cells. This protein was found to localize to cell nuclei, cytosol and processes (inset). **(C)** Exogenous expression of myc-ZNF804A in DD7 CTX0E16-neurons, resulted in the protein localizing to punctate structures along doublecortin (DCX) positive neurites and at growth cones. Yellow dash box highlights magnified images (inset) of neurite processes demonstrating localization of myc-ZNF804A at growth cones. Scale bars = 20 µm **(A + B)** and 10 µm **(C)**.

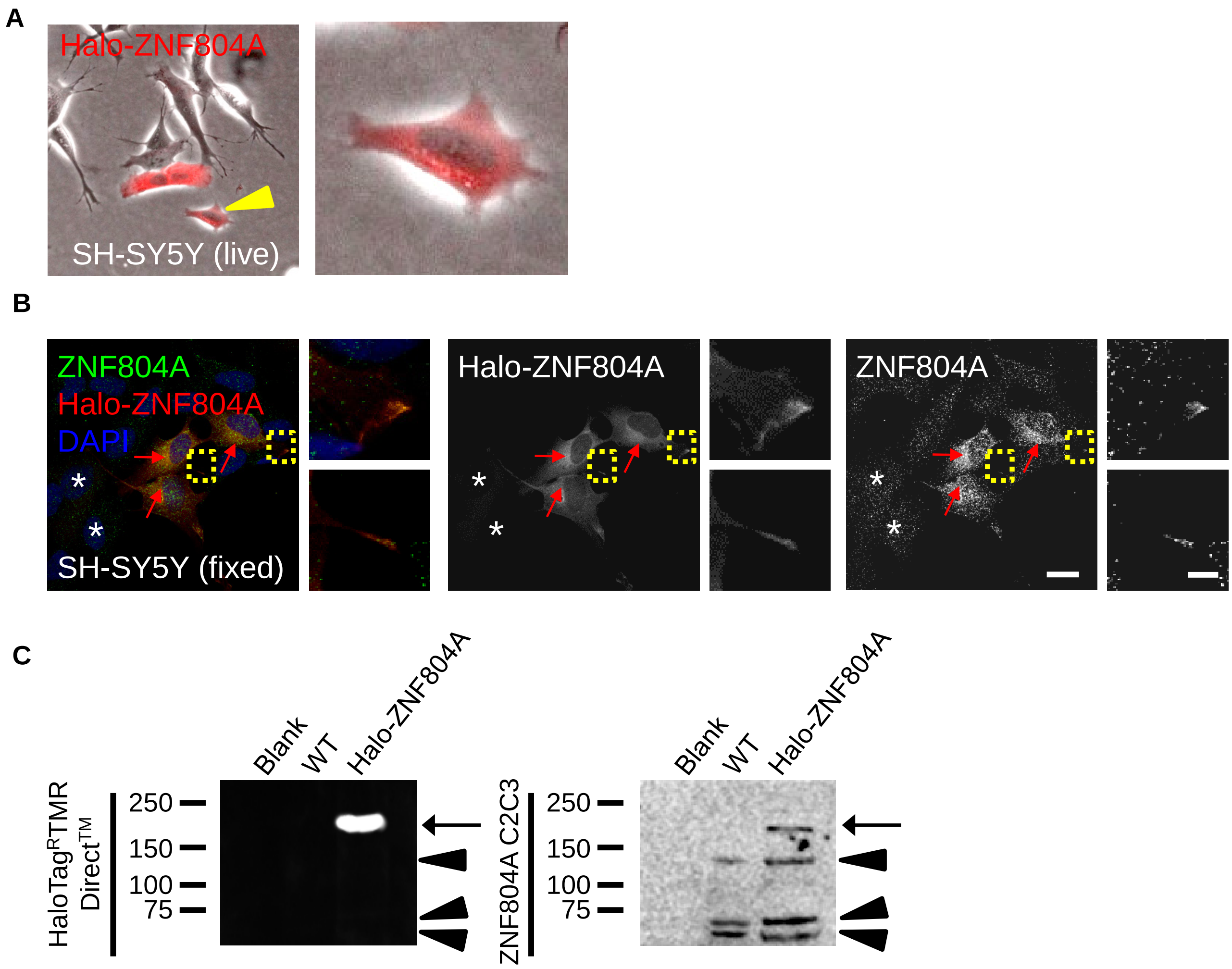

Supplemental Figure S5: Localization of halo-ZNF804A in SH-SY5Y cells. (A) Localization of halo-ZNF804A in a polyclonal population of live SH-SY5Y cells. Halo-ZNF804A was observed using the HaloTag^{R}TMRDirect^{TM} ligand in live cells. Yellow arrow identifies Halo-ZNF804A positive cells, and cell magnified in inset. Halo signal could be observed in cell cytoplasm as well as along plasma membrane. **(B)** Representative confocal image of fixed SH-SY5Y cells overexpressing Halo-ZNF804A. Cells were tripled labelled with the HaloTag^{R}TMRDirect^{TM} ligand, C2C3 antibody, and DAPI. Halo-ZNF804A was observed within the cell nuclei, cytosol and processes (magnified images). Moreover, C2C3 immunofluorescence was strongest in Halo positive cells (red arrow); magnified images demonstrates overlap of C2C3 staining with HaloTag ligand signal. C2C3 immunofluorescence could also be observed in non-transfected cells (asterisks), suggesting that wildtype SH-SY5Y cells express ZNF804A at significant levels. **(C)** Western blot of wild-type and Halo-ZNF804A-expressing SH-SY5Y cell lysates probed with HaloTag^{R}TMRDirect^{TM} ligand and C2C3 antibody. A single band >150 kDa was detected by the HaloTag ligand in Halo-ZNF804A cells, confirming presence of exogenous Halo-ZNF804A. An identical band could also be observed in Halo-ZNF804A-expressing, but not wildtype, SH-SY5Y (arrow). In addition, several smaller proteins bands could be detected in both wildtype

and Halo-ZNF804A SH-SY5Y cell lysates, further indicating expression of endogenous protein (black arrow heads). Scale bars = 20 µm **(B) and** 1 µm **(B, magnified boxes)**.

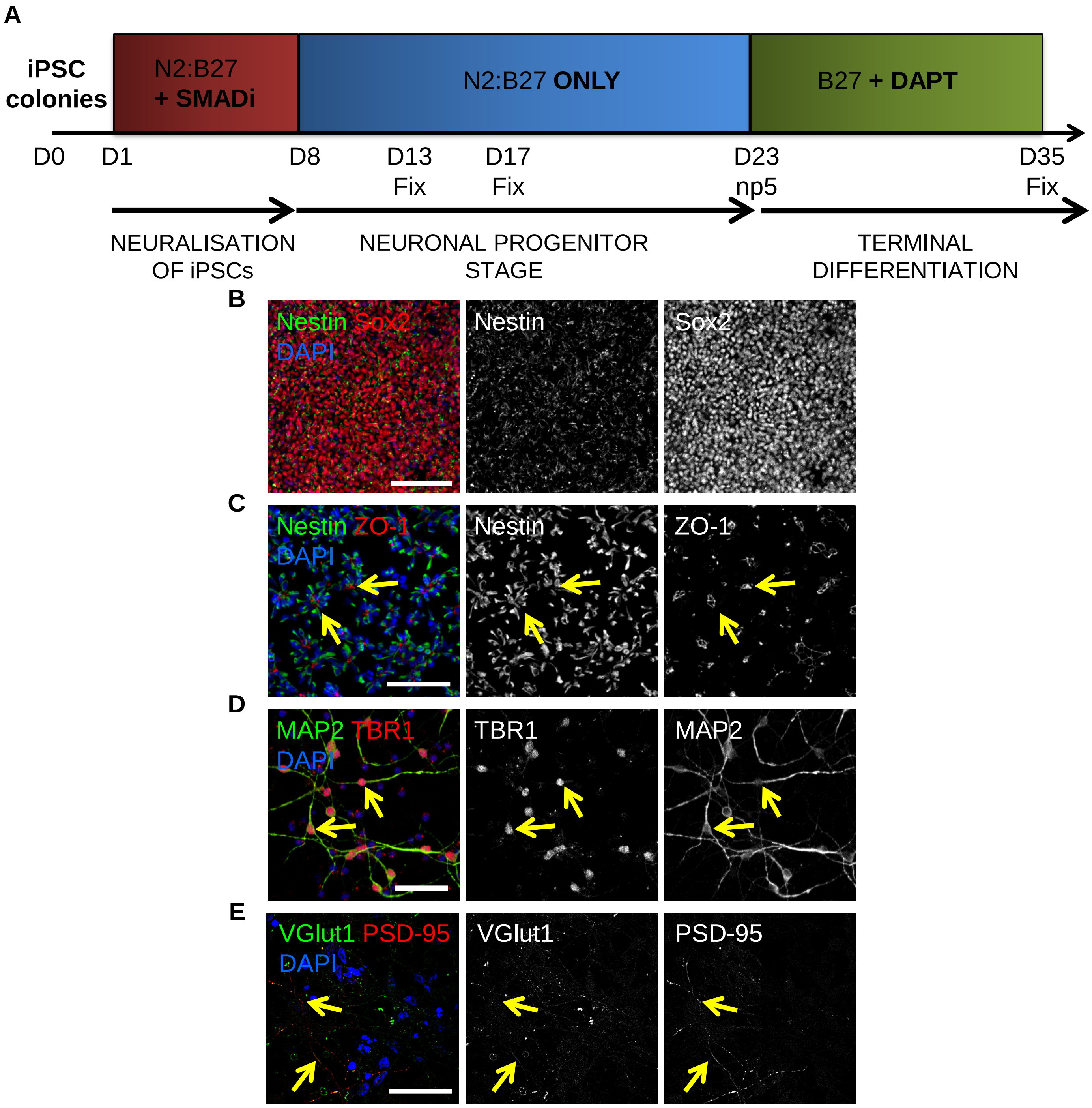

Supplemental Figure S6: Derivation of neurons from human iPSCs. (A) Schematic of neuralization protocol: hiPSCs reprogrammed from keratinocytes of healthy individuals were neuralized as a monolayer in the presence of SMAD inhibitors. **(B)** Following 8 days of neuralization, a population of early neuroepithelial cells were formed, as determined by positive staining for nestin and SOX2. **(C)** Subsequent formation of neural progenitor cells (NPCs) was determined by formation of neural rosettes. Apical lumen of rosettes were positive for ZO-1 with nestin positive cells forming a radial structure surrounding the lumen. **(D + E)** Terminal differentiation of NPCs resulted in the differentiation of projecting

glutamatergic neurons which were positive for TBR1 **(D)** VGlut1 and PSD-95 **(E)**. Scale bar = 100 µm **(B,C)**; 50 µm **(D)** or 20 µm **(E)**.

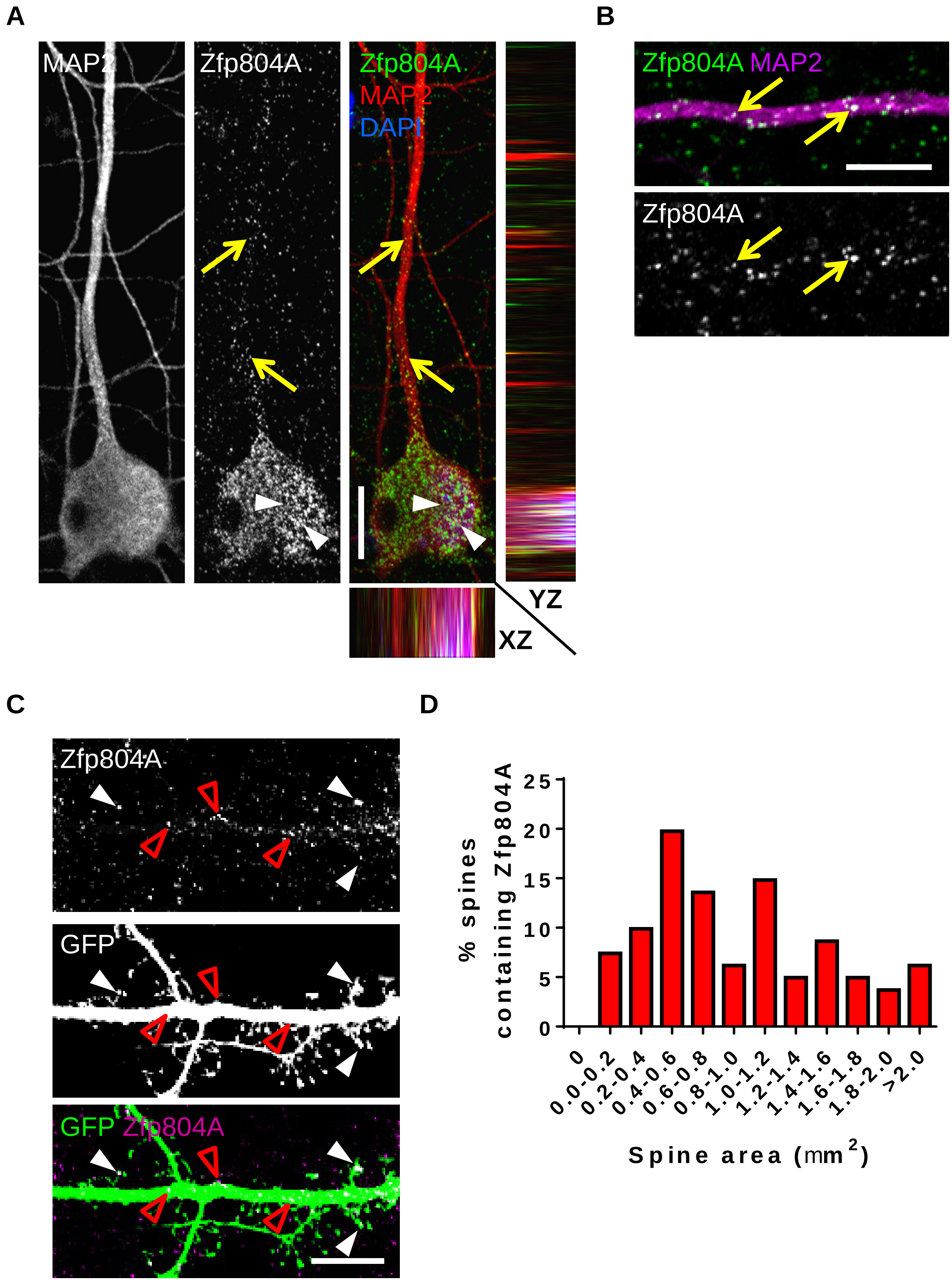

Supplemental Figure S7: Expression of Zfp804A in mature rat primary cortical neurons. (A) Endogenous Zfp804A is present in the nucleus and somato-dendritic compartments in MAP2-positive cortical neurons. Zfp804A was observed within the nucleus (white arrow heads) and as punctate structures along dendrites (yellow arrows). **(B)** Zfp804A puncta are also found along distal MAP2 positive dendrites

(yellow arrows). **(C)** Zfp804A localized to the dendritic shaft (open red arrow heads) and dendritic spines (white arrow heads) in the dendrites of mature primary cortical neurons. **(D)** Histogram of the areas of spines that contain Zfp804A. A larger proportion of spines that contained Zfp804A had small-to-medium area (<1 μm^2), while fewer large spines (>1 μm^2) contained Zfp804A signal. Scale bar = 5 µm **(A, B + C)**.

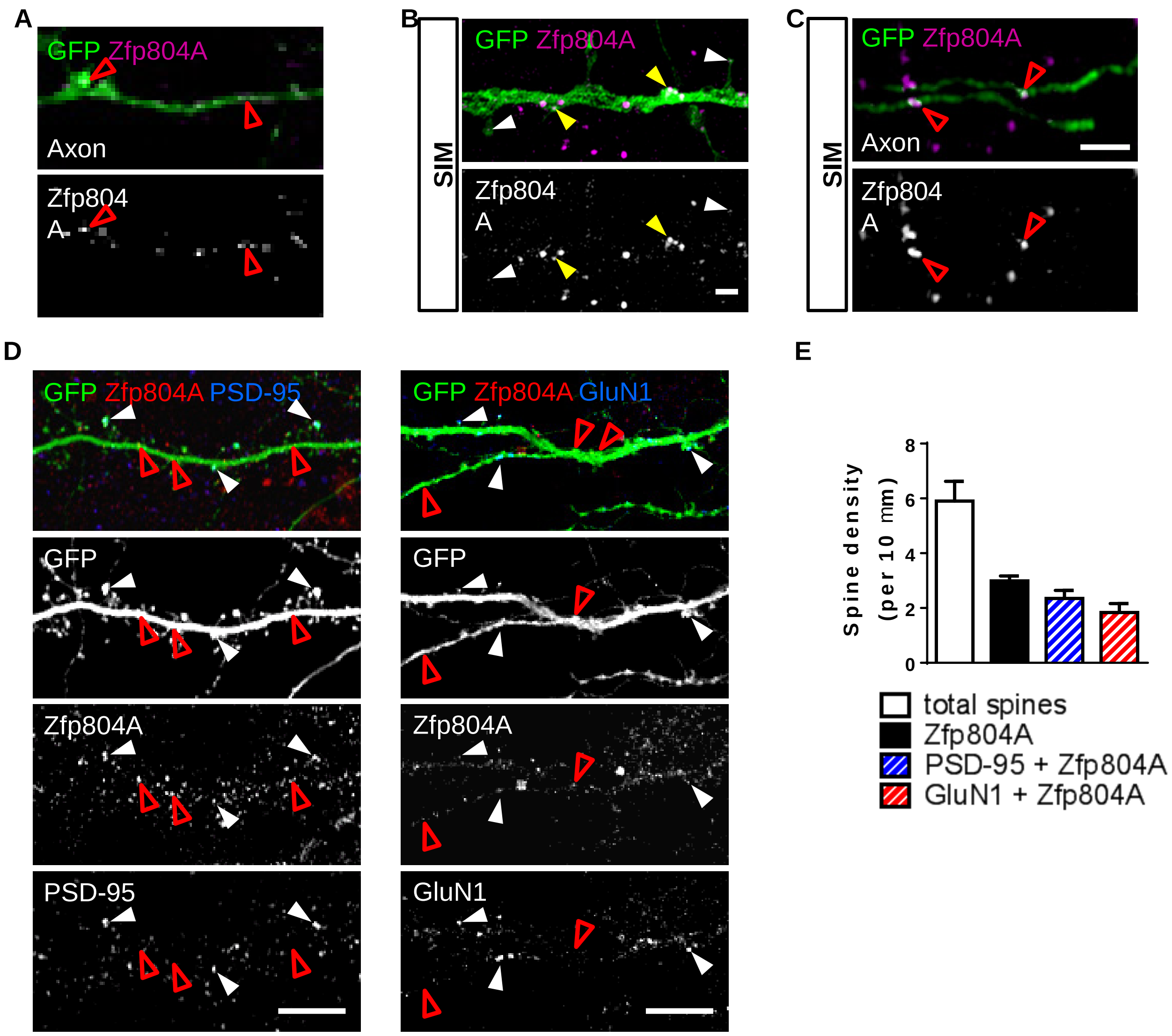

Supplemental Figure S8: Zfp804A is present at synapses in mature primary cortical neurons. (A) Examination of endogenous Zfp804A distribution in DIV 25 cortical neurons revealed that a small amount of protein was present in axon processes (open red arrow heads). **(B)** Superresolution SIM images of Zfp804A sub-cellular localization in GFP expressing primary rat cortical neurons (DIV 25). Zfp804A is present in spine heads (white arrow heads) and along the dendrite (red arrow heads). **(C)** SIM image of axon from GFP-expressing neuron reveals that Zfp804A is present in small puncta along axons (open red arrow heads). **(D)** Representative confocal image of GFP-expressing cortical neurons co-stained for Zfp804A and either PSD-95 or GluN1. This revealed that a fraction of Zfp804A puncta co-localize with PSD-95 and GluN1 in spines (white arrows heads). Open red arrow heads label Zfp804A puncta not co-localized. **(E)** Quantification of Zfp804A, PSD-95, GluN1 and co-localized puncta in dendritic spines (spines per 10 µm: total, 5.9±0.68; Zfp804A, 2.9±0.17; Zfp804A + PSD-95, 2.34±0.32; Zfp804A + GluN1, 1.8±0.23). Scale bars = 5 µm **(A + D)** or 1 µm **(B + C).**

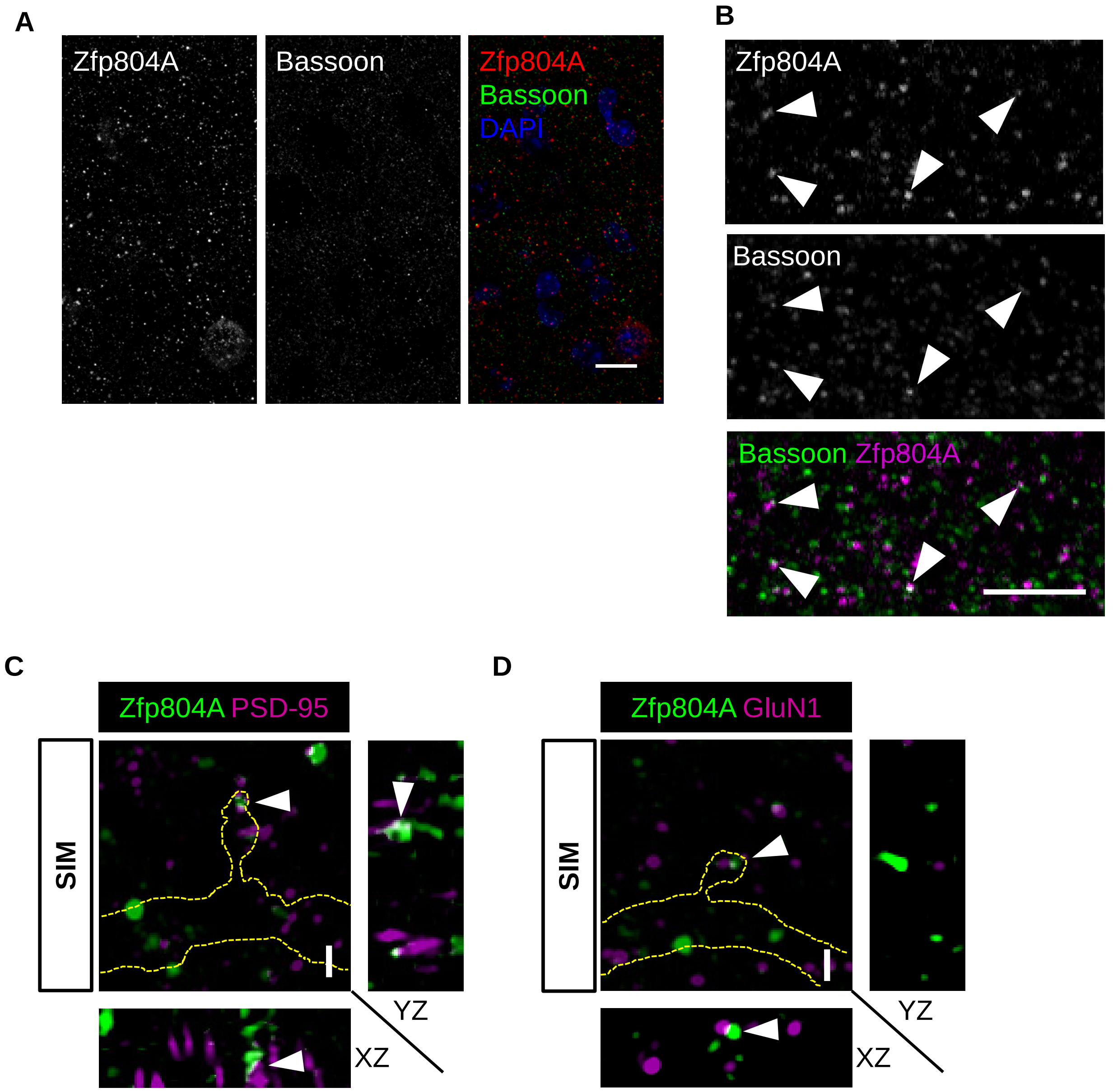

Supplemental Figure S9: Endogenous expression and localization of Zfp804A *in vivo* and subsynaptic distribution of Zfp804A in mature rat primary cortical neurons. (A + B) Representative confocal image of frontal cortex taken from a 6 week old male mouse, triple stained for bassoon (pre-synaptic marker), Zfp804A and DAPI. Zfp804A is present as punctate structures within DAPI-positive nuclei as well as in extranuclear compartments. Magnified insets **(B)** demonstrate that Zfp804A also colocalizes with bassoon (white arrow heads; overlap seen as white) consistent with the synaptic localization of this protein observed in primary cultures. **(C + D)** High magnification SIM image demonstrate partial colocalized of Zfp804A with PSD-95 (**C**) or GluN1 (**D**) puncta (indicated with white arrow heads) within GFP-transfected dendritic spines (outline of spine indicated with yellow dashed lines). Orthogonal views in the XZ and YZ planes confirm colocalization of Zfp804A with

synaptic proteins: colocalization is indicated by white overlap. Scale bars = 50 µm **(A),** 5 µm **(B)** and 500 nm **(C + D)**.

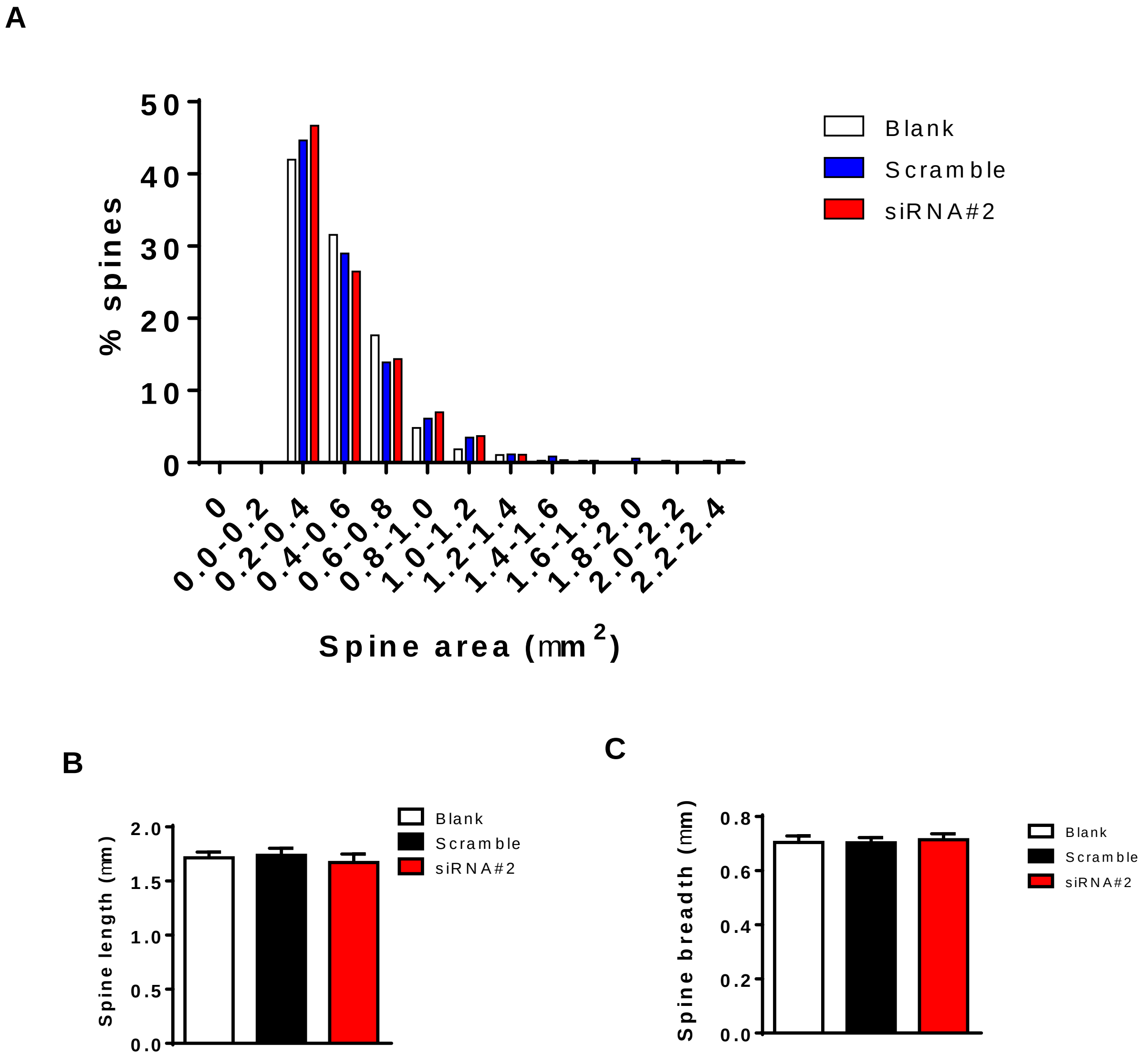

Supplemental Figure S10: Impact of siRNA treatment on dendritic spine morphology in primary rat cortical neurons. (A) Distribution of dendritic spine sizes in rat primary cortical neurons following 5 day treatment with a blank control (no transfection), a scramble siRNA control or siRNA#2. Spine area was not found to be significantly altered in neurons treated with siRNA#2 relative to the two control conditions. **(B)** Quantification of spine length in rat cortical neurons following siRNA treatment. Spine length was not found to be significantly altered in neurons treated with siRNA#2 relative to the two control conditions. **(C)** Quantification of spine breadth in rat cortical neurons following siRNA treatment. Spine breadth was not found to be significantly altered in neurons treated with siRNA#2 relative to the two control conditions.

Supplemental References

1. Pollock K, Stroemer P, Patel S, Stevanato L, Hope A, Miljan E, et al. (2006): A conditionally immortal clonal stem cell line from human cortical neuroepithelium for the treatment of ischemic stroke. *Exp Neurol*. 199:143-155.
2. Stevanato L, Corteling RL, Stroemer P, Hope A, Heward J, Miljan EA, et al. (2009): c-MycERTAM transgene silencing in a genetically modified human neural stem cell line implanted into MCAo rodent brain. *BMC Neurosci*. 10:86.
3. Xie Z, Srivastava DP, Photowala H, Kai L, Cahill ME, Woolfrey KM, et al. (2007): Kalirin-7 controls activity-dependent structural and functional plasticity of dendritic spines. *Neuron*. 56:640-656.
4. Cocks G, Curran S, Gami P, Uwanogho D, Jeffries AR, Kathuria A, et al. (2014): The utility of patient specific induced pluripotent stem cells for the modelling of Autistic Spectrum Disorders. *Psychopharmacology (Berl)*. 231:1079-1088.
5. Nishimura K, Sano M, Ohtaka M, Furuta B, Umemura Y, Nakajima Y, et al. (2011): Development of defective and persistent Sendai virus vector: a unique gene delivery/expression system ideal for cell reprogramming. *J Biol Chem*. 286:4760-4771.
6. Takahashi K, Tanabe K, Ohnuki M, Narita M, Ichisaka T, Tomoda K, et al. (2007): Induction of pluripotent stem cells from adult human fibroblasts by defined factors. *Cell*. 131:861-872.
7. Muller FJ, Schuldt BM, Williams R, Mason D, Altun G, Papapetrou EP, et al. (2011): A bioinformatic assay for pluripotency in human cells. *Nat Methods*. 8:315-317.
8. Chambers SM, Fasano CA, Papapetrou EP, Tomishima M, Sadelain M, Studer L (2009): Highly efficient neural conversion of human ES and iPS cells by dual inhibition of SMAD signaling. *Nat Biotechnol*. 27:275-280.
9. Shi Y, Kirwan P, Smith J, Robinson HP, Livesey FJ (2012): Human cerebral cortex development from pluripotent stem cells to functional excitatory synapses. *Nat Neurosci*. 15:477-486, S471.
10. Srivastava DP, Woolfrey KM, Penzes P (2011): Analysis of dendritic spine morphology in cultured CNS neurons. *J Vis Exp*.e2794.
11. Anderson GW, Deans PJ, Taylor RD, Raval P, Chen D, Lowder H, et al. (2015): Characterisation of neurons derived from a cortical human neural stem cell line CTX0E16. *Stem Cell Res Ther*. 6:149.
12. Gustafsson MG, Shao L, Carlton PM, Wang CJ, Golubovskaya IN, Cande WZ, et al. (2008): Three-dimensional resolution doubling in wide-field fluorescence microscopy by structured illumination. *Biophys J*. 94:4957-4970.
13. Tao R, Cousijn H, Jaffe AE, Burnet PW, Edwards F, Eastwood SL, et al. (2014): Expression of ZNF804A in human brain and alterations in schizophrenia, bipolar disorder, and major depressive disorder: a novel transcript fetally regulated by the psychosis risk variant rs1344706. *JAMA Psychiatry*. 71:1112-1120.

14. Hill MJ, Jeffries AR, Dobson RJ, Price J, Bray NJ (2012): Knockdown of the psychosis susceptibility gene ZNF804A alters expression of genes involved in cell adhesion. *Hum Mol Genet*. 21:1018-1024.
15. Bernstein HG, Steiner J, Dobrowolny H, Bogerts B (2014): ZNF804A protein is widely expressed in human brain neurons: possible implications on normal brain structure and pathomorphologic changes in schizophrenia. *Schizophr Bull*. 40:499-500.
16. Hinna KH, Rich K, Fex-Svenningsen A, Benedikz E (2015): The Rat Homolog of the Schizophrenia Susceptibility Gene ZNF804A Is Highly Expressed during Brain Development, Particularly in Growth Cones. *PLoS One*. 10:e0132456.
